# Environmental risk factors in puppies and kittens for developing chronic disorders in adulthood: A call for research on developmental programming

**DOI:** 10.3389/fvets.2022.944821

**Published:** 2022-12-23

**Authors:** Virginie Gaillard, Sylvie Chastant, Gary England, Oliver Forman, Alexander J. German, Jan S. Suchodolski, Cecilia Villaverde, Pascale Chavatte-Palmer, Franck Péron

**Affiliations:** ^1^Research and Development Center, Royal Canin, Aimargues, France; ^2^NeoCare, Université de Toulouse, Ecole Nationale Vétérinaire de Toulouse (ENVT), Toulouse, France; ^3^School of Veterinary Medicine, University of Nottingham, Nottingham, United Kingdom; ^4^Wisdom Panel, Kinship, Waltham-on-the-Wolds, Leicestershire, United Kingdom; ^5^Institute of Life Course and Medical Sciences of Small Animal Medicine, University of Liverpool, Liverpool, United Kingdom; ^6^Gastrointestinal Laboratory, Department of Small Animal Clinical Sciences, Texas A&M University, College Station, TX, United States; ^7^Expert Pet Nutrition, Fermoy, County Cork, Ireland; ^8^Université Paris-Saclay, Université de Versailles Saint-Quentin-en-Yvelines (UVSQ), Institut National de Recherche Pour l'Agriculture, l'Alimentation et l'Environnement (INRAE), Biologie de la Reproduction, Environnement, Epigénétique et Développement (BREED), Jouy-en-Josas, France; ^9^Ecole Nationale Vétérinaire d'Alfort, BREED, Maisons-Alfort, France

**Keywords:** behavior, epigenetics, microbiota, nutrition, obesity, chronic enteropathy, developmental programming

## Abstract

Many dogs and cats are affected by chronic diseases that significantly impact their health and welfare and relationships with humans. Some of these diseases can be challenging to treat, and a better understanding of early-life risk factors for diseases occurring in adulthood is key to improving preventive veterinary care and husbandry practices. This article reviews early-life risk factors for obesity and chronic enteropathy, and for chronic behavioral problems, which can also be intractable with life-changing consequences. Aspects of early life in puppies and kittens that can impact the risk of adult disorders include maternal nutrition, establishment of the gut microbiome, maternal behavior, weaning, nutrition during growth, growth rate, socialization with conspecifics and humans, rehoming and neutering. Despite evidence in some species that the disorders reviewed here reflect the developmental origins of health and disease (DOHaD), developmental programming has rarely been studied in dogs and cats. Priorities and strategies to increase knowledge of early-life risk factors and DOHaD in dogs and cats are discussed. Critical windows of development are proposed: preconception, gestation, the suckling period, early growth pre-neutering or pre-puberty, and growth post-neutering or post-puberty to adult size, the durations of which depend upon species and breed. Challenges to DOHaD research in these species include a large number of breeds with wide genetic and phenotypic variability, and the existence of many mixed-breed individuals. Moreover, difficulties in conducting prospective lifelong cohort studies are exacerbated by discontinuity in pet husbandry between breeders and subsequent owners, and by the dispersed nature of pet ownership.

## Introduction

There is increasing awareness that aspects of early life in puppies and kittens, especially nutrition during gestation and early growth, impact the risk of neonatal mortality (1–3) and the development of chronic diseases in adulthood (4, 5). In many mammalian species, early-life and parental experiences have been investigated as potential contributors to the developmental origins of health and disease (DOHaD). The concept of DOHaD encompasses the observations that environmental exposures during development can drive epigenetic changes that modify, or “program” the expression of genes, affecting structural and functional development, with rapid or delayed risks to health. In humans, the first 1,000 days of life, which approximates to gestation plus 2 postnatal years, have been identified as a critical period when developmental programming sets the foundations for optimal neurodevelopment, growth and health (6). Much of what is known about DOHaD and the epigenome is derived from laboratory animal models (7), but experimental knowledge has also accrued for ruminants, pigs and even horses (7–9).

In dogs and cats, most research into the etiology of chronic adulthood conditions has focused on adult environmental predictors and risk factors, without investigating whether these have developmental origins. Literature searches in PubMED^®^ (31 July, 2022) with the broad search string (epigenetics OR “developmental programming” OR DOHaD OR “developmental origins of health and disease”), combined with “Dogs” or “Cats” as Medical Subject Headings, retrieved 218 articles. After screening titles and abstracts for relevance, 51 articles relating to dogs and 6 relating to cats remained; the most apparent topics of interest were epigenetic modifications in cancer cells [32 articles (56%)], and epigenetic aspects of breed phenotype. Overall, there has been inadequate consideration in dogs and cats of the extent to which the environment, during different stages of growth and maturation, can influence the subsequent occurrence of adult conditions and behavioral traits, even if these environmental factors are chronologically distant. Research in domestic carnivores has been led mainly by experts in specialized fields of veterinary medicine, including nutrition, reproduction, gastrointestinal microbiology, and behavior, with a paucity of expertise in DOHaD that crosses the relevant disciplines.

This review provides an overview of the main environmental risk factors in puppies and kittens that can affect the occurrence of obesity, chronic enteropathy (CE) and behavioral problems in adulthood. These chronic disorders are common in domestic carnivores, challenging to treat, and have major deleterious effects on health, quality of life and potentially longevity (10–12). Difficult behavior can lead to a break-down in the human–animal bond, and may result in abuse, relinquishment or euthanasia of pets (13–15). It is possible that some of the modifiable variables explored may represent ongoing risks that commence or become apparent in early life, and some may be manifestations of DOHaD, with changes in the epigenome at periods of developmental plasticity. There is also the potential for exposures to unmask the effects of DOHaD. Suggested research priorities are discussed for each condition, based on existing research in puppies and kittens, factors in adult dogs and cats known to be associated with the condition and hypothesized to become established during early life, and on knowledge of developmental programming in other species. Research strategies are proposed to increase our understanding of the long-term impact of early environment and life events for dogs and cats. Such strategies must include studies to determine the role of DOHaD as has been done for other species. These studies might ultimately allow the generation of guidelines to inform disease prevention from as early as preconception. This is not only important for animal welfare, but should be considered in the broader economic and societal context of dog and cat ownership.

Building upon evidence in dogs and cats, humans and laboratory animals, we propose a timeline of key exposures and developmental milestones in puppies and kittens that shape and define “early life.” Early life in this review is not intended to relate to a fixed chronologic age or necessarily to the same period of development classically considered in DOHaD studies in other species. It is used to describe the periods preceding adulthood in which the physiological and psychological maturation of puppies and kittens can be affected for good or bad, or modifiable risk factors for later chronic disease emerge. This is intended to help frame future research and to encourage breeders, owners and veterinarians to take a holistic, integrated and proactive approach to promoting the long-term health of pets (10–12).

## The context for research on early-life development of dogs and cats

### Societal

Dogs and cats are cherished as family members in many households, making their long-term health a high priority for owners. Societal benefits of dog and cat ownership include the promotion of human health and wellbeing (16, 17); dogs also work in a wide variety of service roles. While these factors, combined with a general concern for animal welfare, provide a rationale for advancing our understanding of early-life risk factors for chronic diseases, they also mean that acceptance of invasive research in these species is limited; this is likely to be one reason for the relatively slow advancement of DOHaD knowledge in dogs and cats.

With respect to large-scale observational studies, the dispersion of the pet population makes studying connections between early and late exposures and events particularly challenging. There is no coherent network of the relevant parties throughout a pet's life. For example, each individual dog or cat may have a different breeder who is responsible for its prenatal and first 2–3 postnatal months of life, and each pet may subsequently be homed with a different owner, who in turn may have a different veterinarian.

### Economic

The size of the pet population and the direct economic significance of pets are tangible measures that help to contextualize the importance of pursuing avenues for preventive medicine. There are an estimated 92.9 million dogs in Europe (25% of households; 2021 data) (18), and 83.7–88.9 million in the USA (45% of households; 2020 data) (19). The total population of cats is estimated to be more than 113.6 million in Europe (26% of households; 2021 data) (18) and 60.2–61.9 million in the USA (26 % of households; 2020 data) (19). Sales of pet food products were €27.2 billion in Europe in 2021 (18). In the USA in 2021, market sales were $50.0 billion for pet foods and treats, $34.3 billion for veterinary care and product sales, and $9.5 billion for other services outside of veterinary care, such as boarding, grooming and insurance (20).

### Biological

#### Breed

Large phenotypic variability within the canine species, and to a lesser extent the feline species, contributes to the complexity of research in companion animals. The canine species exhibits the widest morphological and weight differences between breeds of all terrestrial mammalian species. More than 350 breeds of dogs are recognized by the International Cynological Federation (21). Adult weights range from 1 kg, for a Chihuahua, to more than 100 kg, for an English Mastiff. Moreover, many pet dogs (up to 40% in the UK) are a mix of breeds (22). Age at which adult body weight is attained correlates with dog breed size, ranging from ~9 to 10 months for toy, small and medium-sized breeds, to 11–15 months for large and giant breeds (23). Size diversity is less pronounced in the cat population, in which 45 breeds are recognized (24) and only 5–15% of cats are pedigreed (25). Adult cat weights range from ~2 kg for a Munchkin to 10 kg for a Maine Coon (25).

#### Reproductive biology

Understanding early-life risk factors for adult diseases and the potential for developmental programming requires a knowledge of species-specific biology of conception and fetal and neonatal development (summarized for dogs and cats in Supplementary Figure 1, Supplementary Tables 1, 2). This allows the timing of environmental exposures to be related to the differentiation of cell types and the development of specific tissues and organs. Overall embryonic and fetal development is similar between dogs and cats (26) with the exception of oocyte maturation and ovulation. Ovulation in cats is typically induced by coitus (26), although spontaneous ovulation seems to be more than anecdotal (27). Oocytes are released in metaphase II, so fertilization can occur as soon as they reach the oviduct (28). In dogs, there is spontaneous ovulation of immature oocytes at prophase I. Oocyte meiosis resumes after ~ 48 h in the oviduct, and fertilization occurs from 90 h after ovulation (28, 29). Another difference in dogs is that follicles undergo preovulatory luteinization, so serum progesterone concentrations are already high at ovulation (28, 29).

#### Milestones of early life

Dogs and cats share many major biological milestones with other species, but the timing and biological details differ. These milestones include embryonic and fetal events (Supplementary Figure 1; Supplementary Tables 1, 2), neonatal survival, transition to solid foods and neutering. Periods of organ and organ/system development and maturation in which external factors can modify its developmental trajectory are numerous. These critical periods represent different windows of opportunity to promote development beneficial to long-term health.

## Early-life environmental exposures and events as risk factors for selected disorders in adult dogs and cats

### Obesity in dogs and cats

As stated by Kopelman (2000), “obesity can be defined as a disease in which excess body fat has accumulated such that health may be adversely affected” (30). Obesity is defined as a chronic relapsing disease, which itself can predispose to other non-communicable diseases, such as diabetes mellitus, cardiovascular diseases and cancer in dogs and cats (31). In the field of veterinary medicine, over 20 national and international veterinary and associated organizations support the classification of obesity as a disease (32), which is regarded as the number one health problem in companion animals (33).

**Figure 1:**
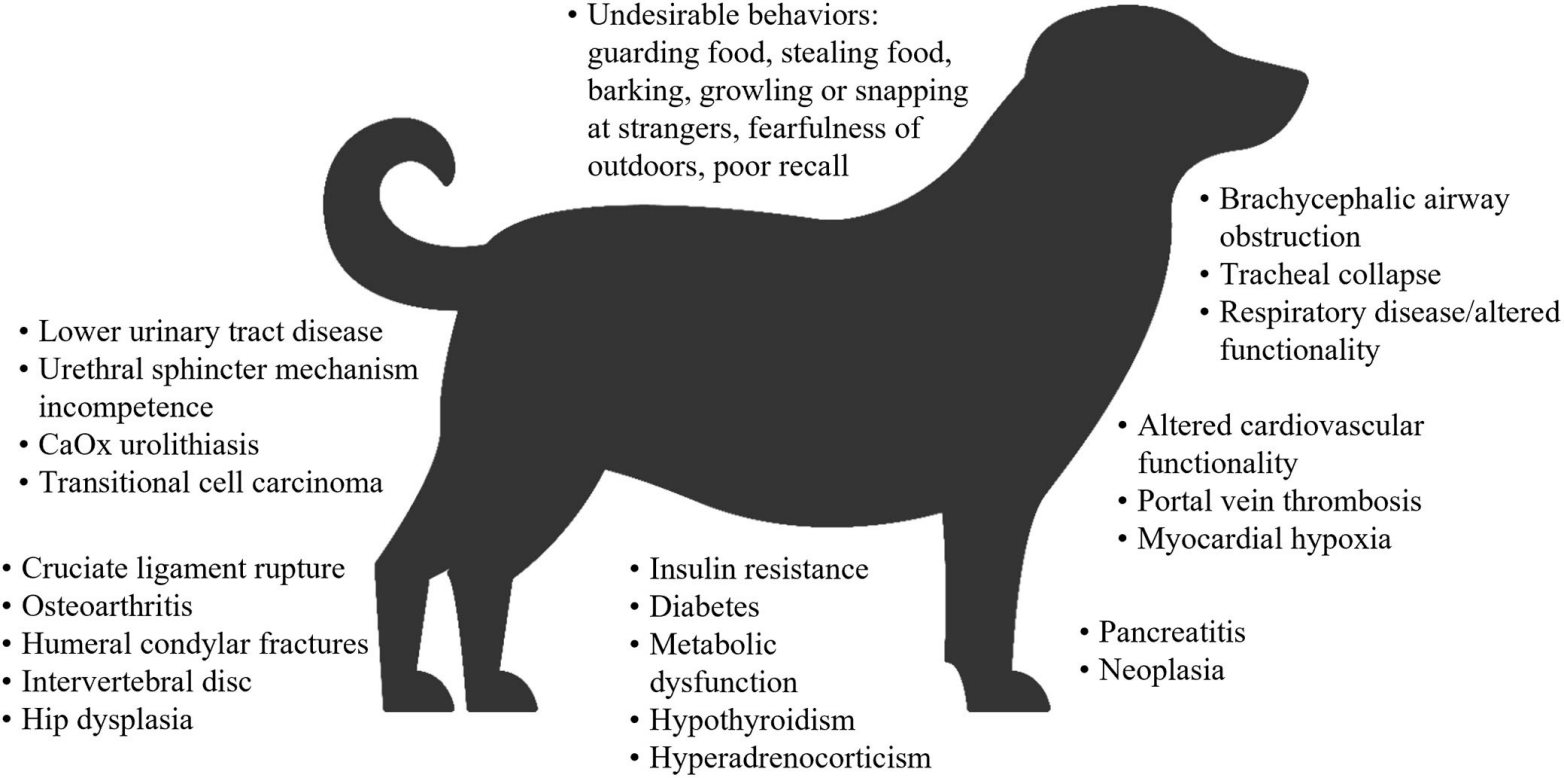
Overview of comorbidities associated with obesity or overweight in dogs (31, 37–40, 42, 43, 46–49). Some associations were specific to either obese dogs or overweight dogs. CaOx, calcium oxalate.

**Figure 2:**
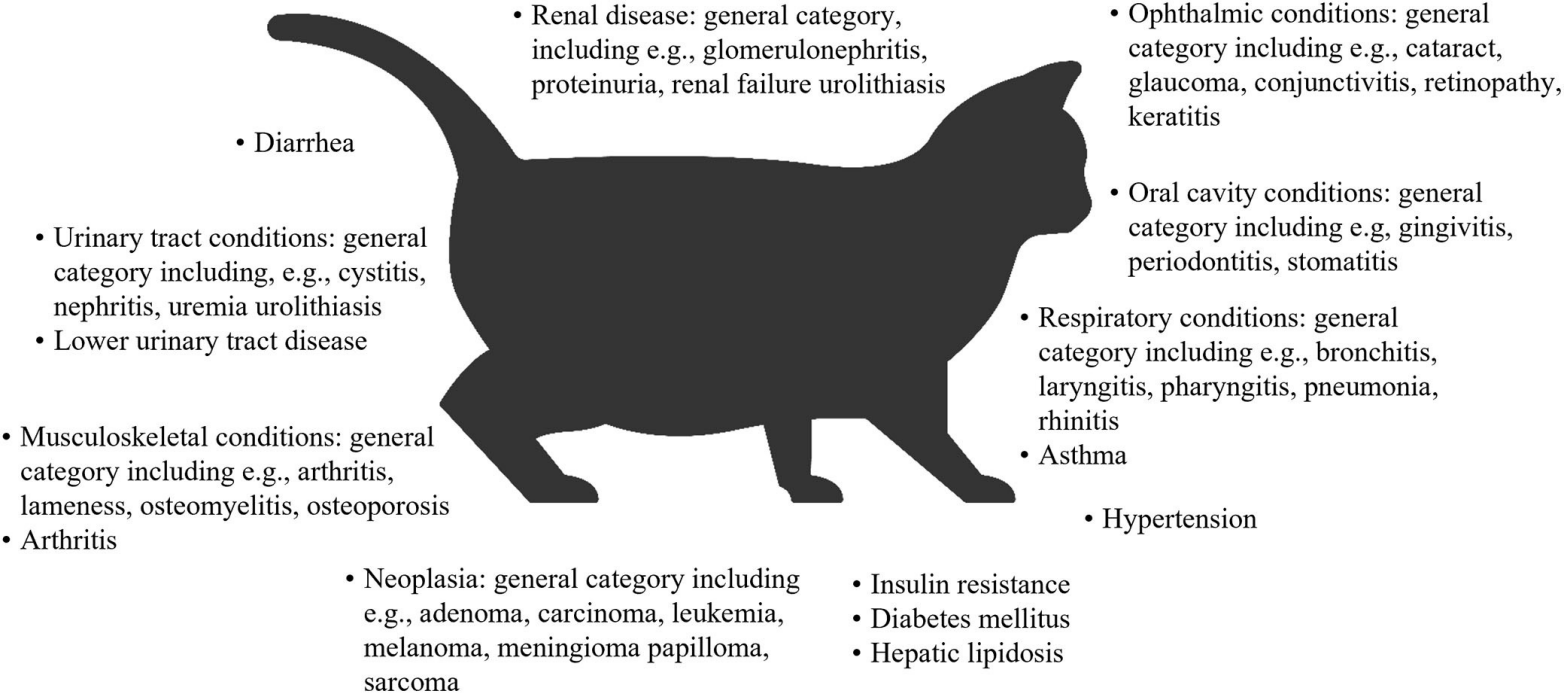
Overview of comorbidities associated with obesity or overweight in cats (31, 52, 53, 56–58). Some associations were found only for certain body condition scores within the obese or overweight range. Associations were often found between obesity or overweight and a general category of disease or conditions pertaining to an organ/organ system. In these cases, examples are provided of some of the conditions that the study included in the category definition.

Overweight and obesity in both dogs and cats is generally measured by determining the body condition score (BCS), which correlates well with adipose tissue mass (32–35). On this basis, a study of dogs at family pet shows in the UK reported that 65% of adult dogs were overweight or had obesity, and 9% had obesity (10). In the 2018 obesity prevalence survey in the USA conducted by the Association for Pet Obesity Prevention (APOP), veterinarians assessed 36.9% of dogs as overweight, and 18.9% as having obesity (36). Obesity and/or overweight in dogs are associated with many comorbidities, functional impairments (37–43), a shorter lifespan (44), and a poorer quality of life (45) (Figure 1). In the APOP 2018 survey for cats, the prevalence of overweight and obesity were 26 and 34%, respectively. The prevalence of overweight or obesity in adult cats at vaccination visits in New Zealand was 22 and 3%, respectively (50). As for dogs, overweight and/or obesity in cats is associated with an increased risk of a wide range of co-morbidities (46, 51, 52), a reduced lifespan and a higher risk of death (severe obesity only) (53, 54), and some, but not all data, suggest a reduced quality of life (55) (Figure 2).

#### Early-life risk factors for obesity in adult dogs

Risk factors for obesity can be identified in early life, as early as the fetal period. For example, low birth weight in Labrador Retrievers has been associated with overweight in adulthood even after adjusting for age and neuter status: 70% of dogs with birth weights below the median were overweight as adults, compared with 47% of dogs with birth weights above the median (5). No association was found between adult obesity and growth rate between birth and Day 2 or between Day 2 and Day 15. In contrast, in a study of female Beagle colony dogs raised in controlled environmental conditions, birth weight did not correlate with adult overweight status, but fast growth rate from birth to 2 weeks was a predictor for adult overweight at 2 years of age (4). By the age of 7 months, BCS discriminated between dogs that would be overweight as adults and those that would be slightly overweight or ideal weight (4). No significant difference was found between adult weight groups in their energy intake or resting energy expenditure corrected for metabolic bodyweight at the age of 4 months. Resting energy balance between the age of 7 and 10 months was significantly higher in puppies who were overweight compared with ideal weight in adulthood. During this study, dogs were fed *ad libitum* (time-restricted after weaning) with a diet formulated for growth, or for neutered adults, as appropriate. In a different study, a retrospective analysis of veterinary practice records found that dogs that were obese by 3 years of age (127 breeds, 93% neutered) had faster growth in body weight between 12 and 60 weeks of age than that modeled in healthy dogs in ideal body condition (59).

Neutering is common for dogs and cats and is often performed before puberty while they are still developing. The exact timing varies between countries, species and breeds, and is still a controversial issue. Neutering is well established as one of the most significant risk factors for obesity in adults (46, 60–65). Prepuberty or peripuberty neutering in the context of this review is considered an early-life environmental exposure of relevance to the risk of obesity, albeit during the later stages of development. The potential impact of sex (60, 66, 67) and age of neutering on the effects of neutering on adult obesity are unclear because findings differ by study. A prospective cohort study of Golden Retrievers identified an ~ 42% greater risk of obesity in dogs neutered between 6 and 12 months of age compared with those neutered at >1 year of age, but no difference in risk between neutering at <6 months compared with 6–12 months and >1 year (66). Conversely, in a retrospective study of veterinary records, age at neutering (ages ≤ 6 months, >6 months to ≤ 1 year and >1 to ≤ 5 years of age) was not associated with the risk of obesity (67). When growth patterns of pet dogs from the same proprietary data source were examined, neutering before and after 37 weeks was associated with slight upward or downward shifts in growth trajectory, respectively; however, these shifts were small, suggesting limited overall impact on weight gain and, therefore, future obesity (68). Differences in study design and dog breeds might explain apparent inconsistencies between these studies. Indeed, interactions between breed size, age at neutering and number of veterinary visits per year were reported to affect the risk of overweight (67).

Hormonal changes resulting from neutering could have a direct effect on the risk of obesity. Neutered dogs have lower metabolizable energy requirements than sexually intact dogs (69, 70), and neutering can increase indiscriminate appetite (71). Evidence suggests that neutering could also unmask or augment the effects of environmental exposures in the younger animal. Increases in the bodyweight of female Beagle dogs after neutering between the ages of 7 and 10 months were higher in dogs retrospectively identified to be at risk of adult obesity by having a higher neonatal growth rate than their contemporaries of an ideal adult bodyweight (4). The growth of Labrador Retrievers between 2 and 21 days of life was associated with risk of obesity at adulthood, but only in neutered dogs (72).

#### Early-life risk factors for obesity in adult cats

In humans, breast feeding has a protective effect against childhood obesity compared with feeding formula milk (73), which might in part be associated with the presence of leptin in breast milk. Leptin is a hormone produced by adipose tissue that inhibits food intake and modulates glucose metabolism; the main source for neonates may be maternal milk (74). In a small study in cats, the odds for overweight in adulthood were 3 times less in kittens suckled for >6 weeks compared with <6 weeks, and there was a predisposition for overweight with suckling duration of 11 weeks or less (75). The investigators hypothesized that a short suckling period might lead to perturbations in the development of control mechanisms for fat accumulation and body composition through curtailment of leptin intake. A fast growth rate in cats is a key risk factor for obesity. A comparison of *ad libitum*-fed colony cats that were overweight with those of ideal weight at a median of 8.5 years of age, showed a significant association between growth rate between 3 and 12 months and later overweight status (76). In a different study that modeled the growth of colony cats fed *ad libitum* from weaning, early growth rate indicated by weight at 15 weeks of age was a significant predictor of being overweight at 9 years (77). Hypotheses to explain these associations include genetic, epigenetic and *in utero* factors, in addition to physical activity, food quality, feeding behavior and the gut microbiome (76).

Faster growth rate, smaller litter size, lower birthweight, and maternal overweight before pregnancy were associated with a predisposition of kittens to be overweight at 8 months of age in a study focused on genetic factors, and designed to reduce the potential for non-genetic confounders and epigenetic differences (78). Despite the study design, some of the findings suggested that developmental programming might have played a role. The authors speculated that epigenetics might underlie the weak but significant negative correlation of litter size with overweight at 8 months. Also, epigenetic differences might have contributed to the observation that, although both overweight mothers vs. lean and variable-weight mothers, and male vs. female sex were associated with faster weight gain of kittens, this relationship became statistically significant for the maternal phenotype later than the sex difference.

As in dogs, neutering of male and female kittens is a risk factor for adult obesity (65, 79, 80). Whilst neutering is associated with increased appetite and food intake (81–83), it is also associated with reduced maintenance energy requirements (70, 81, 84). There is insufficient evidence in cats to know whether the age at neutering is associated with risk of obesity. However, differential changes in appetite have been associated with age of neutering; acute hyperphagia was observed in female cats neutered at 31 weeks of age but not in those neutered at 19 weeks of age (85). These behavioral changes may be associated with the effects of neutering on appetite-related hormones such as ghrelin, leptin, adiponectin and glucagon-like peptide-1 (86). For example, in a study of adult male cats, serum concentrations of adiponectin rapidly decreased after neutering, and within 7 days, there was a significant increase in serum concentration of ghrelin (83).

#### Potential research priorities

##### Nutrition

The role of early-life nutrition in the development of adult obesity demands more extensive and diverse research. In puppies and kittens and their parents, nutrition is relatively easy to modify in both research and “real-world” settings, and is likely to have multiple impacts on factors associated with obesity (87–91). At its simplest, chronic excessive calorie intake that starts at a young age results in progressive accumulation of body fat that ultimately manifests as adult obesity. However, a wealth of evidence in other species, including humans, shows that nutritional insults both *in utero* and postnatally can program later obesity and other metabolic disorders (92, 93). Models of obesity in polytocous species demonstrate that poor maternal nutrition (quantitative and/or qualitative) can modulate aspects of fat deposition and energy homeostasis in offspring through epigenetic mechanisms (89, 94, 95). Alterations in the development of the offspring's hypothalamus-adipose tissue axis are believed to be particularly important for obesogenic traits, manifested as structural changes, mal-programming of appetite regulation favoring orexigenic pathways, central leptin and insulin resistance, and alterations in noradrenergic innervation of adipose tissue (96, 97). Research is needed to determine if low birth weight in puppies, as a risk factor for adult obesity, is an example of fetal programming of a “thrifty” phenotype, whereby a metabolic profile set to cope with inadequate nutrition during pregnancy, later becomes a risk factor for obesity in the context of abundant postnatal nutrition. Paternal nutrition in laboratory animal models can also program obesogenic traits in the offspring (98), but this does not appear to have been researched yet in companion animals.

Obesogenic traits can also be sensitive to postnatal nutritional environment as development of organs and hormonal pathways continues after birth in mammals (87). The literature on postnatal maturation of domestic carnivores is limited, and as in other species, the timing depends on the organs involved (Supplementary Tables 1, 2). For example, changes in the morphology of organs such as the adrenal gland can occur during the first year (99), functional maturation of digestive processes may not occur until 3 months (100) and the immune system may not attain all adult characteristics until 12 months (101). Myelination of the neocortex continues to increase until ~9 months after birth (102). Nevertheless, it is reasonable to hypothesize that developmental plasticity is concentrated in the suckling period. Research is needed in dogs and cats to determine the effects of diet in the pregnant and lactating dam on the quantity and quality of colostrum and milk, and whether these effects have consequences for the offspring's adult body composition and metabolism. The evaluation of the impact of food intake and nutritional interventions of the first days of life is particularly relevant for low birth weight puppies and kittens when considering nutritional interventions; rapid catch-up growth is associated with an increased risk for adult obesity in other species (97).

##### Growth

In both puppies and kittens, higher growth rates have been associated with adult obesity (59, 77). It is unclear if and how aspects of energy balance regulation during growth predispose adults to be obese or of ideal weight. Postprandial decreases in acylated ghrelin, an orexigenic gut hormone, are delayed in 7-month old female Beagles already identified as being on a trajectory to adult overweight, and this may promote excess food intake (4). The basal plasma concentration of leptin is positively associated with adiposity but does not appear to be an early predictor of weight gain. In humans; evidence suggests that leptin's main role is to signal low body fat stores in situations of negative energy balance (4, 103). Research is needed in larger study populations with different breeds and sexes to characterize further the dynamics of energy balance during growth associated with adult obesity, and to evaluate any role of developmental programming and the environmental triggers. Ideally, studies of growth and obesity should evaluate body composition. However, whilst for practical reasons BCS is most commonly used to evaluate adiposity, the scoring scales have only been properly validated in adult dogs and cats. Puppies and kittens have different body composition profiles and morphologies compared with adult dogs and cats (104–106), which makes diagnosing overweight status with BCS scales designed for adults unsatisfactory. Greater objectivity and more uniformity between studies might easily be achieved by evaluating growth against growth rate standards now available for a comprehensive range of different breed sizes from 12 weeks of age (68, 107). These standards will be valuable in facilitating DOHaD research in obesity by identifying rapid or slow growth at an early age, and for case ascertainment in body composition and metabolic studies.

##### Gut microbiota

Differences in the gut microbiota and/or microbiome of obese vs. lean adult dogs and cats have been observed (108–112) and changes characterized in obese dogs and cats during diet-driven weight-loss studies (108, 113, 114). However, associations between diet, gut microbiota, enteroendocrine hormones and metabolic disturbances are complex, with studies reporting contrary findings (113, 115, 116). When the effects of macronutrient ratios in diets fed to both dams and their kittens were evaluated, composition of the pre-weaning diet did not affect the profile of bacterial populations in kitten feces at 8 weeks, but did modulate expression levels of genes in the glucose and metabolic pathways in blood samples taken at 18 weeks (117). The findings were reversed for a comparison between two post-weaning diets. What has not been investigated directly is any association between gut microbiota as it is developing in the puppy and kitten and adult obesity, and the potential for early nutrition to influence this. Research in other species on developmental programming suggests that could be a fruitful avenue of research (118, 119).

In mice, gut microbiota mediate changes in global histone acetylation and methylation of DNA both locally in cells of the colon and distally in tissues such as liver and white adipose tissue (120). These microbe-mediated changes have been demonstrated in species other than dogs and cats during early life at a time when the gut microbiota is developing (121). Microbial metabolites have a direct role in epigenetic modifications, and the composition of the gut microbiota is relevant because the profile of metabolic byproducts of dietary constituents such as short-chain fatty acids (SCFAs) may differ between bacterial species (120, 122). Factors such as suckling vs. bottle feeding, lifestyle, environment and exposure to antimicrobials may also impact obesogenic traits through their effects on the emergent microbiota (123, 124).

##### Neutering

The strength of neutering as a modifiable risk factor for obesity in both dogs and cats demands a greater understanding of the interactions between sex hormones and diet on appetite-related hormones and blood metabolites (86). The impact of neutering at different stages of development (early vs. late) needs to be dissected to resolve differences between studies and explore sex, species and breed differences. The impact of environmental exposures such as nutrition and growth rate during the first days/weeks/months on the effects of subsequent neutering is under-researched, but existing data warrant further longitudinal prospective studies (4). One question to be addressed is whether neutering unmasks or potentiates the effects of developmental programming puppies or kittens.

##### Interaction of environmental exposures with genetic susceptibilities to overweight and obesity

The interaction of genetic risk factors with modifiable variables in development can increase or decrease the likelihood of particular phenotypes. In humans, genes enriched or only expressed within the central nervous system have a central role in the biology of obesity (125). Knowledge of genetic susceptibilities can help researchers design studies on developmental programming and interpret their results.

Dog breeds including Pug, Beagle, Golden Retriever, English Springer Spaniel, Border Terrier, Labrador Retriever, and Cavalier King Charles Spaniel are at a higher risk for overweight than crossbred dogs (126, 127), whilst domestic short-hair cats have an increased risk of obesity (127). Candidate genes for genetic variants suspected to increase the risk of obesity in dogs include *POMC, FTO, PPARG, MC4R, and MC3R, INSIG2, GPR120* (127). Genetic variants may be restricted to a small number of breeds, e.g., a 14 base-pair deletion in *POMC* associated with obesity and food motivation found in Labradors and Flat-coated Retrievers (128, 129). Genetic risk factors need to be a consideration in studies investigating the impact of early-life environment on obesity. Genome-wide association studies could help elucidate the genetic background of obesity in companion animals and there is potential value in both within breed and large-scale across-breed approaches.

### Chronic enteropathy in dogs and cats

Chronic enteropathy is an overarching term that encompasses subgroups of chronic intestinal disorders based on treatment response: immunosuppressant-responsive enteropathy [IRE, previously known as idiopathic inflammatory bowel disease (IBD)], food-responsive enteropathy, and antibiotic-responsive enteropathy (12). The prevalence of CE reported in different studies ranges from 1 to 18% (12). In cats, IRE frequently coexists with small cell lymphoma (130), which is considered to fall under the umbrella of CE in this species (131). Although the underlying etiology of each subtype of CE is unclear, and may not be the same, they are chronic inflammatory conditions, and the pathogenesis reflects interactions between the gut microbiota and gut immune systems in the context of environmental factors such as diet, and genetic susceptibilities in some breeds.

#### Early-life risk factors for chronic enteropathy in adult dogs

There are very few data on early-life risk factors for CE in dogs; however, these limited data implicate a diverse range of variables that warrant full investigation. Puppies that had historically presented in the acute stages of canine parvovirus infection at a median of 12 weeks of age, had a greater risk of owner-reported chronic gastrointestinal signs in later life than control dogs that presented at the veterinary clinic either for a routine check or for signs not associated with parvovirus [odds ratio 5.33 (95% CI: 2.12–14.87)] (132). A similar study also found that previous parvovirus enteritis was a risk factor for persistent gastrointestinal signs, and among dogs that had recovered from parvovirus infection, markers of disease severity were associated with that risk (133). In another study, early modifiable risk factors for CE in adulthood included vaccination of the dam during pregnancy, type of solid food fed to puppies during the first 6 months, and the puppy's body condition (“slim” rather than “normal weight”) (134). These results should be interpreted with caution because of methodological limitations such as retrospective owner questionnaires, participant bias and broad diet types that were not nutritionally controlled. In a retrospective review of veterinary records from a medical teaching hospital in the USA, neutering was associated with an increased odds of IBD in males and especially female dogs (odds ratios for neutered vs. sexually intact 1.43 and 2.0, respectively, *p* < 0.05 for both) (135). The authors hypothesized that the same anti-inflammatory and antioxidant effects of estradiol demonstrated in murine models could be protective against IBD in dogs.

#### Early-life risk factors for chronic enteropathy in adult cats

Although chronic enteropathy in cats is well described in the literature (131, 136), no studies were found that have investigated modifiable risk factors in kittens.

#### Potential research priorities

Disruption to the maturing gut microbiota, which might be due to diet or antimicrobials, is associated with increased risk of later IBD in humans or experimental colitis in animal models (137). Research suggests that epigenetic modifications underlie interactions between diet, the immune system and the microbiota in the development of chronic diseases including IBD (121, 138).

**Figure 3:**
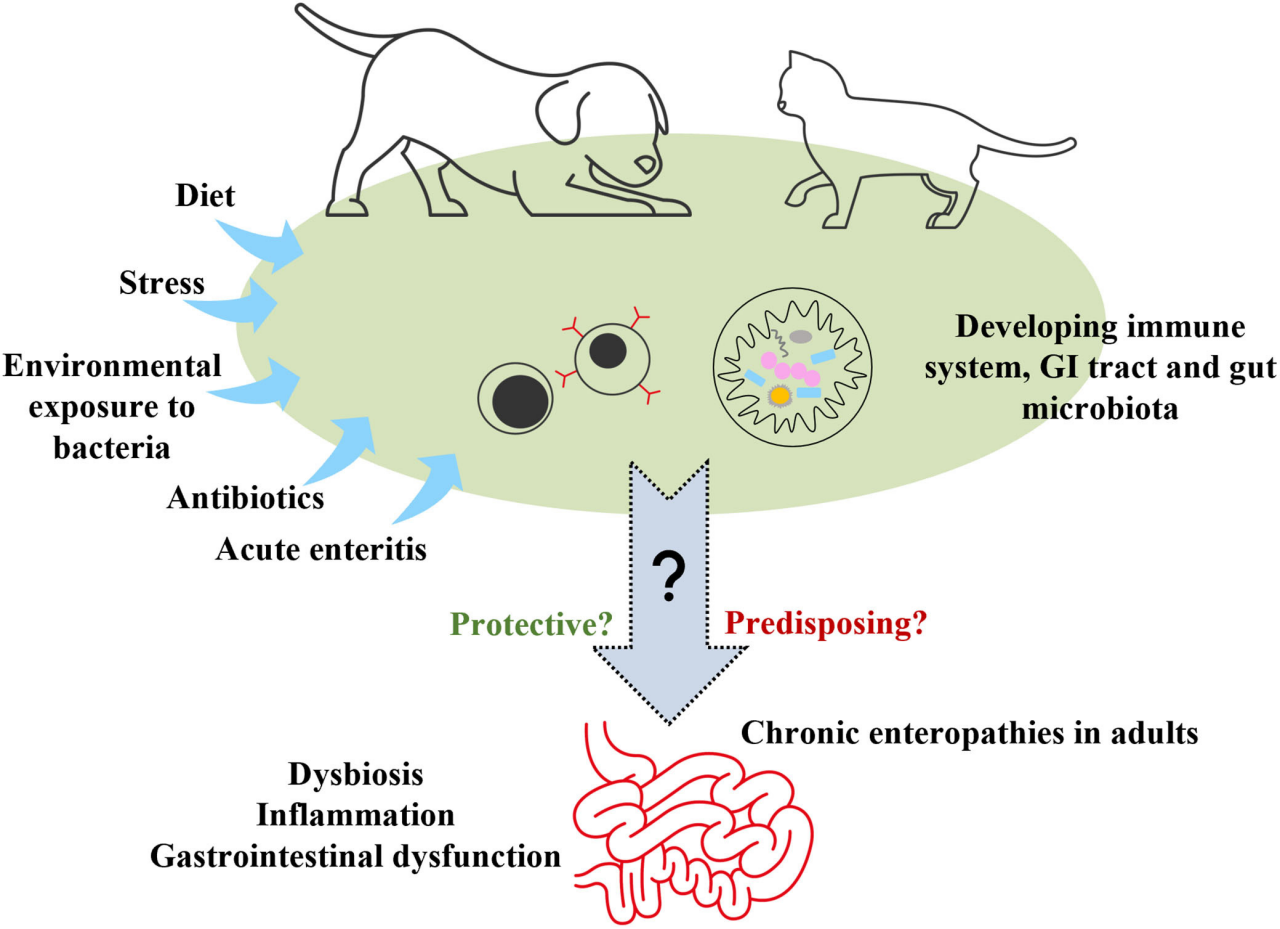
Early-life environmental exposures to investigate as potential risk factors for chronic enteropathies in adulthood. GI, gastrointestinal.

We suggest that investigation of any association between gut microbiota in puppies and kittens and development of CE in adulthood should be a research priority (Figure 3). There are complex interrelationships between gut microbiota, host metabolism, the immune system, intestinal inflammation and gastrointestinal health or dysfunction (139). Is there a relationship between the gut microbiota that develops in puppies and kittens and that found in adult dogs and cats with CE, which differs from that in healthy adults? Do perturbations in the developing gut microbiota affect the maturation of the immune system and acquisition of tolerance in ways that predispose puppies and kittens to later CE? Do the effects of microbiota dysbiosis on the gut metabolome in these pets epigenetically program susceptibility to future CE and/or dysbiosis? Pieces of the puzzle have been characterized in puppies and kittens (140, 141), and separately in adults with CE (139, 142, 143), but the existence of a link between these has not yet been established.

Bacterial dysbiosis is defined as alterations in the composition of the bacterial gut microbiota leading to functional changes in the microbial transcriptome, proteome or metabolome, and/or decreased bacterial diversity (139, 144, 145). It is reported that 72%−79% of dogs and 76% of cats with CE have dysbiosis as evaluated by dysbiosis indices (142, 146, 147). Research in humans and animal models suggest that the role of dysbiosis in the pathogenesis of IBD could be causative (148, 149). This makes the development of the gut microbiota in puppies and kittens, and perturbations of this, of particular interest as a potential risk factor for CE.

The possibility in dogs of intra-uterine bacterial transfer from dam to fetus is controversial, but after birth, data suggest that the dam seeds the initial bacteria and her individual microbial profile plays a fundamental role in shaping the gut microbiota of her litter (150). The richness of bacterial species in the neonatal gut increases from day 2 after birth, and the gut microbiota changes significantly with age during the suckling and weaning period (151). The greatest changes to the microbiota of healthy puppies had occurred by 5–6 weeks of age in one investigation (151), although differences between the microbiota of offspring and dam were still apparent at 8 weeks in another study (152), and small changes might feasibly occur until 1 year of age (153). As with puppies, the gut microbiota of healthy kittens develops substantially during suckling and weaning, although the adult profile might not be fully achieved in those periods (154, 155). In a study in kittens, changes in the microbiome were still evident at 18–30 weeks of age, but had stabilized by 30–42 weeks (156). In another study, the microbiome was relatively stable in kittens aged 8–16 weeks (141, 157). Further longitudinal investigation is clearly needed. It is contended that the microbiota established in puppies and kittens is likely to be generally stable during healthy adult life as for humans, but this needs to be verified (139, 151, 152).

The relevance of dietary influences on gut microbiota and the gut microbiome in puppies and kittens to later gastrointestinal health status is likely to be multifactorial. Existing research needs to be extended to investigate early diet as a potential risk or protective factor for CE. For example, pre- and probiotic supplementation of Great Danes in the last week or last 4 weeks of pregnancy reduces the risk of neonatal gastroenteritis in their offspring (158). It is hypothesized that this protective effect is conveyed *via* the entero-mammary link, given that in other studies feeding dams with pro and/or prebiotics improved the immune properties of their colostrum (159, 160). Another hypothesis (not mutually exclusive) is that the effect is mediated by the selection of health-promoting bacteria in the dam that then colonize the neonates.

The most profound disturbances to gut microbiota are those caused by antibiotic use. It is hypothesized that antibiotic use could be a major priming factor in puppies for later CE. In humans, antibiotic treatment in the first postnatal year is associated with an increased risk of later development of IBD (161–163). Acute diarrhea is common in puppies, and it is often treated with antibiotics. The fecal microbiota changes in dogs with acute diarrhea and the bacterial groups involved are not consistently reported to be the same as in chronic diarrhea, although reduction in fecal concentrations of SCFAs is a shared finding (139, 145, 164). In a prospective controlled study, metronidazole (a common antibiotic treatment for acute diarrhea) significantly altered the fecal microbiome and metabolome of healthy dogs, including a decrease in the abundance of Fusobacteria, which are key SCFA-producing bacteria, and the main bile acid converting bacterium *Clostridium hiranonis* that was associated with a reduction in secondary bile acids (165). Changes persisted in nearly half of the dogs for at least 4 weeks. The long-term effects of such treatment in puppies still establishing a normal gut microbiota needs to be explored in studies on developmental programming. A course of antibiotic treatment (20 or 28 days) in 2-month old cats with upper respiratory tract disease was shown to delay the maturation of their gut microbiota compared to healthy untreated cats (166). The duration of effects differed between antibiotics; the impact of amoxicillin-clavulanate on the microbiome occurred mainly during treatment, whereas the impact of doxycycline was observed from 1 to 3 months after antibiotic withdrawal (166). Research should extend to the use of antibiotics in pregnant dogs; data in humans and mice suggest that this is a risk factor for gastrointestinal disease in the offspring (167, 168).

It is not known whether the gut microbiota influences early development of the gastrointestinal tract and susceptibility to chronic disease through epigenetic modifications in puppies and kittens. Data from mouse studies however, point to the importance of gut microbiota in modulating post-natal development of the gut through DNA methylation of genes in intestinal epithelial cells associated with immunity, metabolism, and vascular regulation (122, 162). Changes in bacterial metabolites associated with CE in dogs are known in other species to influence epigenetic modifications affecting immune and inflammatory pathways. For example, decreased fecal abundance of *Fusobacterium* and *Faecalibacterium* in dogs with CE is associated with reduced fecal concentrations of the SCFA propionate (147, 169). Short-chain fatty acids can regulate epigenetic modifications by inhibiting histone deacetylases (HDACs) and contributing acetyl donors for DNA or histone modifications.

Abnormalities or deficiencies in immune responses to environmental antigens, together with genetic susceptibilities, appear to play central roles in the development of CE in dogs (170). The possibility that some of the immunopathogenesis in dogs with CE has origins in epigenetic changes was raised by an investigation of the reduced intestinal expression of mucosal IgA found in these dogs (171). Hypermethylation of the gene for TACI was negatively associated with expression of mucosal IgA; the authors hypothesized that such changes in methylation status might have been induced by inflammatory mediators and exposure of the gut to an altered intestinal microflora (171). Such mechanisms might therefore be a link between environmental exposures during development and risk of later CE.

Across all the avenues of research suggested, developmental periods of particular interest in puppies and kittens include initial colonization of the neonatal gut, weaning, and the transition from breeding facilities to new owners, when diarrhea is common, coinciding with changes in diet, stress and exposure to different microbial environments. Large populations need to be studied to understand interindividual variations in microbiota—there may not be a single “normal,” “healthy” microbiota. Robust studies are needed that use nutritionally specific diets and record only veterinarian-diagnosed CE. Those conducting research in developmental programming must of course consider breed susceptibilities and breed-independent genetic associations with disease. Dog breeds susceptible to CE include Weimaraner, Rottweiler, German Shepherd, Border Collie and Boxer (12, 172).

### Behavioral problems in dogs and cats

Behavioral problems in dogs and cats are common and can affect their welfare and quality of life (173), their relationship with humans, and their suitability for assistance work (174). Difficult behavior is frequently cited by owners as being at least one of the reasons for them relinquishing their pets to animal rescue centers, being the primary reason for 10% of dogs in a recent Canadian study (13), and the sole reason for 27% of dogs and 19% of cats in a US study (14). They can also drive some owners to seek elective euthanasia for their pets (15). Although the nature of behavioral problems is wide ranging, such as aggression toward humans and other animals, separation anxiety, and soiling in the house, at least some adverse behavior traits detrimental to the long-term future of dogs and cats can be attributed to their early-life environment.

Most neurological development occurs during fetal life; it continues rapidly in the neonate, but myelin formation and maturation continues until at least 36 weeks of age in dogs (175). Regions of the brain develop at different rates throughout early life, potentially therefore remaining susceptible to environmental exposures (175–177). The development of behavioral and cognitive traits can be considered in different phases: gestation, the neonatal period including feeding, neurological stimulation and mothering in the first 3 weeks, early socialization from ~ 3 to 12 weeks of age, late socialization from 12 weeks up to 6 months, and the enrichment period, which may extend to 1 year of age (177, 178). It is believed that experiences during each period have cumulative effects on trainability, health and performance (177, 178).

#### Early-life risk factors for behavioral problems in adult dogs

No research in dogs investigating the effects of maternal stress or diet during pregnancy on the behavior of offspring was identified, except a mention that puppies of malnourished dams were extremely nervous in addition to displaying physical abnormalities (179).

Poor maternal care and socialization before 3 months of age have been associated with fearfulness in dogs, and poor maternal care alone was also associated with a combination of fearfulness, noise sensitivity and separation anxiety (180). These data were derived from a survey of owners, but other studies with more objective measures show that the level of mothering can affect the performance of dogs in cognition tests, stress responses, and temperament in later life. However, some research findings appear to be contradictory as to whether an environmental exposure has a positive or negative effect. For example, in one prospective study, guide dogs that had experienced more intense mothering had poorer problem-solving abilities and showed higher levels of anxiety at 14–17 months of age, both of which were associated with a significantly greater risk of failing the guide dog training program (174). In contrast, a benefit of greater maternal care was demonstrated in male and female Beagle puppies; the mean duration of daily maternal care in their first 3 weeks was positively correlated with exploration and latency of the first yelp, and negatively correlated with stress in isolation tests at 8 weeks of age (181). A study with long-term follow up found that a higher level of maternal care of male and female German Shepherd dogs was associated with greater physical and social engagement (e.g., ball retrieval, positive acceptance of handling) as well as aggression in young adults at 18 months of age (182). In summary, stimuli in the suckling period appear to effect some behaviors of adult dogs, but the direction of reported associations is not always intuitive or consistent, perhaps reflecting the complexity of the biology as well as interstudy differences in behavior tests, ages, and breeds (181).

In a review of seven observational studies on dogs originating from high-volume commercial breeding establishments and sold either online or through pets shops, risk factors were highlighted for later behavioral and psychological problems (183). In the largest of these seven studies, UK dogs acquired from sources such as pet stores and the internet were 1.8 times more likely to show aggression toward humans than dogs acquired directly from breeders (183, 184). Across studies, aggression was the most common problem behavior associated with commercial breeding establishments or puppy farms and pet stores. Although causative factors were not investigated, potential causes discussed included stress in the dam, insufficient or excessive neonatal stimulation, early weaning and maternal separation, and social isolation between the age of 3 and 12 weeks.

By the early socialization period, the central nervous system has developed to a stage that allows conditioning and associated learning (177). Socialization of puppies with familiar conspecifics is important for the development of communication competency, and early interactions with non-familiar conspecifics may influence the risk of aggressiveness in adult life (177, 185). For example, restriction of a puppy's contact with conspecifics in the 8 weeks after their first exposure to other dogs in a public setting was found to be associated with aggression toward unfamiliar dogs when they were 1–3 years old (185). Early socialization with humans is important for later responses to handling, leash training and stress tests (186).

Behavioral traits and non-social cognitive abilities continue to develop in puppies during the late socialization and enrichment periods (187–189). In young candidate working dogs, measures of inhibitory control, attention and spatial cognition improved between 3 and 12 months of age (187). In a second longitudinal study, performance of cognitive tasks improved between the age of ~ 9 weeks and 21 months, and the adult phenotype for some traits could be predicted from test results in puppyhood (188). However, little is known about specific exposures in these periods that might influence the course of brain development, and the general environmental context, breed and sex are also likely to play a role (189). Questionnaires completed by foster carers of puppies from ~ 2 months of age until the initiation of formal guide-dog training, showed a positive behavioral effect of growing up in a household with another dog and with more experienced puppy raisers (189). Puppies that had been attacked or threatened by an unfamiliar dog showed significantly higher “dog-directed fear” and “stranger-directed aggression” at the age of 12 months old compared with puppies that had not experienced that trauma and had worse training outcomes (189). However the age at which the trauma had occurred was not specified.

A link between epigenetic changes and human-directed social behavior in dogs was found in one study (190). The DNA methylation of the promoter region of the oxytocin receptor gene (*OXTR*) was measured by bisulfite pyrosequencing followed by methylation-specific PCR in mouth epithelial cells obtained from various Canidae. Four differentially methylated 5′-cytosine–phosphate–guanine_3′ (CpG) sites were identified. They were subsequently studied in a large population (*n* = 217) of Border Collies. Not only did DNA methylation status differ between females and males, it was also associated with their response in a “threatening approach” test in a sex-dependent manner. For example, more methylation at a specific CpG site in female dogs tended to correspond with a greater likelihood of appeasing behavior in the test, whereas the opposite relationship was found in male dogs. In addition, CpG sites differed in whether promoter methylation increases or decreased *OXTR* expression levels (190), both neuter status itself and the interaction of sex with neuter status did not predict methylation levels at the three CpG sites investigated. This study highlights the complexity of relationships between epigenetic modifications and behavior in dogs, and the need for research on environmental factors that influence the epigenetics of the *OXTR* gene.

#### Early-life risk factors for behavioral problems in adult cats

There is a dearth of knowledge on the effect of the maternal environment during pregnancy and subsequent behavioral problems in adult feline offspring. However, one study showed that when the kittens of dams that had been malnourished throughout pregnancy were fostered onto non-food deprived cats, both physical and behavioral development were delayed (179, 191). The behaviors affected included time spent playing and the use of a litter tray. Moreover, in adulthood the cats displayed marked antisocial behavior and alternation between dominant and submissive behaviors. These observations could potentially represent classic DOHaD in cats. In other research, protein restriction of cats during late gestation and lactation adversely affected the attachment processes in both dams and kittens in the first 12 days after birth (192).

There is ongoing neurological development of kittens during the first 3 postnatal months (193). This is observed, for example, in increasingly sophisticated ability to respond to sound, development of visual placing and binocular coordination, and gross behaviors during interactions with siblings (193). Early life experiences of cats affect behavior in adulthood (194). For example, human handling of kittens during the sensitive period for socialization has been associated with more friendly behavior toward humans at the age of 1 year (195, 196). The extent to which epigenetic modulation and/or genetic differences contribute to these observations is unknown.

#### Potential research priorities

We suggest that research into the risk factors for behavior problems in dogs and cats needs to diversify to include assessments *in utero* and more studies of environmental influences in animals aged 6–12 months. This should not be at the expense of further work in neonates when brain development is particularly plastic. Neonatal studies are facilitated by the relative ease with which the environment can be controlled for related individuals in the same litter, although this does not allow for the distinction between genetic and environmental effects. Apparently contradictory results for the effect of maternal care on stress responses need to be explored, perhaps by comparing different stress challenges and intensities at various ages in animals kept in standardized environments. More long-term studies are required to determine the durability and reversibility of the effects of early-life environment on adult behavior.

The extent to which epigenetic modulation drives risk factors for behavioral disorders in dogs and cats is not known. The few existing data linking epigenetic changes to dog behavior (190) highlight the complexity of the relationships involved. Environmental factors influencing known epigenetic variation of *OXTR* are especially important to explore. Polymorphisms in the gene for the dopamine receptor 2 (*DRD2*) are associated with fearful behavior in some breeds of dog (197), and a variant haplotype in this gene is associated with anxiety separation in Golden Retrievers (198). *DRD2* could therefore be another gene of interest to study for epigenetic changes affecting behavior.

Studies in other species provide a rich source of developmental behavioral data and hence hypotheses for dogs and cats. Various cognitive, behavioral and emotional disturbances in children have been associated with stress during development (199, 200). Prenatal stress can result in structural and functional changes in multiple regions of the developing fetal brain, including the hypothalamus-pituitary axis (199, 201). DNA methylation of the glucocorticoid receptor gene and *OXTR* are examples of mechanisms believed to link childhood experiences with psychiatric disorders and temperament, respectively (202, 203). Preconception experiences of parents may also be relevant and can affect anxiogenic responses of their offspring and subsequent generations of descendants (204, 205). Rodent studies highlight the importance of the exact timing of environmental exposures and sexual dimorphism in the developmental sequelae (206). Stresses that may affect the behavior of offspring include, for example, preconception psychological trauma of either parent, maternal diet during pregnancy, early separation of offspring from their dam, and mothering behaviors (201, 205, 207, 208). Evidence from various species also links the composition of the gut microbiota with neurocognitive and behavioral development, building the concept of a microbiome-gut-brain axis (138, 209, 210).

## Strategies for research to understand early-life risk factors for chronic diseases and behavioral problems in dogs and cats, and the potential role of developmental programming

The developmental origins of health and disease have not yet been confirmed in pets, but the examples discussed suggest that developmental programming is likely to be as important as in other species. We recommend a concerted multidisciplinary approach to explore developmental programming in dogs and cats and to close the large knowledge gaps compared with other species.

**Figure 4:**
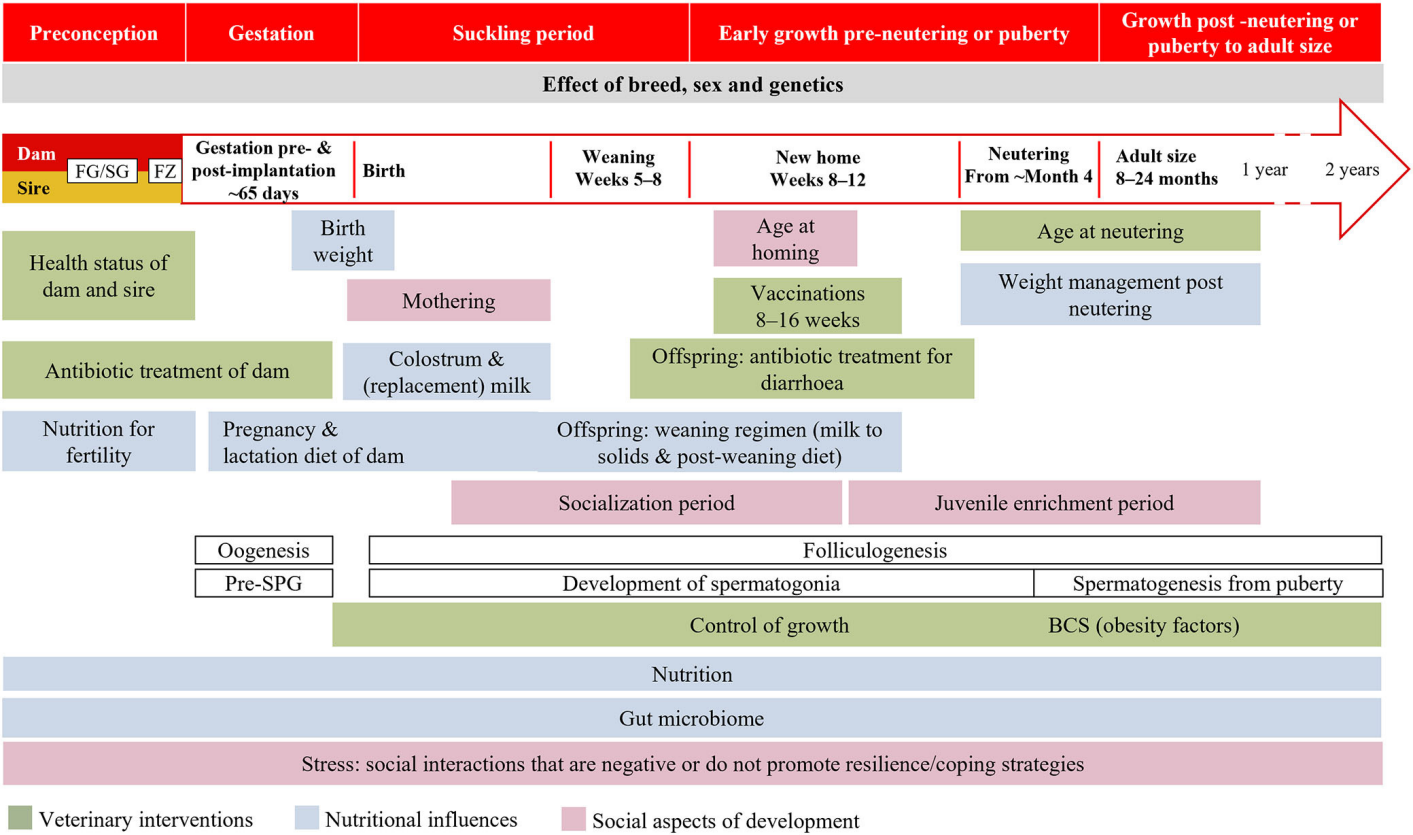
Windows of developmental programming proposed in dogs and cats. BCS, body condition score; FG/SG, folliculogenesis/spermatogenesis; FZ, fertilization; Pre-SPG, prespermatogonia.

Human research and information campaigns on DOHaD are conducted in the context of a species-specific critical window of development. We believe that the overall critical window of time implicated in the development of dogs and cats extends from preconception to the end of growth, comprising five periods: preconception, gestation, the suckling period, early growth pre-neutering or pre-puberty, growth post neutering or post puberty to adult size (Figure 4). Within this window, there will be different and sometimes overlapping critical periods for different aspects of health and disease according to the developmental plasticity of the relevant tissues. The upper age limit of this “window” will depend upon species and breed. Variables of particular interest for DOHaD research in dogs and cats, both individually and in combination, include the maternal and neonatal environment, nutrition and associated weight gain and/or growth rate, the gut microbiota, weaning stress, and neutering (Figure 4). Work in other species highlights the need for studies to consider the periconception period (211), the exact timing of environmental exposure, differences in programming between the sexes (212–214), the role of the placenta (215), paternal influences on the offspring's epigenome, breed and genetic variation (216–218).

It is critical for the whole scientific research community to be able to access large datasets encompassing high quality “whole of life” data and biobanks of tissue samples in order to explore the long-term impact of exposures in early life. Research colonies of cats and dogs with internal breeding programs are useful to address the effect of individual interventions, but they are uncommon and do not reflect the situation of household pets, which are exposed to many non-controlled, interacting environmental factors. Analysis of large prospective observational cohorts of privately owned animals, perhaps spanning up to 15 years to encompass whole lifespan, may identify effects of programming that are small in the individual and very variable between individuals. A particular challenge to be addressed is obtaining longitudinal data for a dog or cat that follows both parents in the preconception period and extends through gestation and neonatal life to adulthood and end of life. This might need data from two breeders (one for the sire and one for the dam), at least one owner, and probably at least three veterinary surgeons (one each for the sire, dam and puppy or kitten). These stakeholders need to be engaged with the potential wide-ranging and long-term implications of DOHaD and the many possible opportunities for intervention. Institutions that breed and train dogs for service roles, and subsequently monitor their progress, e.g., guide dogs, can provide collaborative opportunities for researchers.

Although there are ongoing large, prospective and observational longitudinal studies, these primarily target dogs after they have left the breeder, and so will lack data from the first 2 months after birth. For example, the Generation Pup project operated by the Dogs Trust in the UK was initiated in 2016 to use owner and veterinary data from up to 10,000 dogs to identify modifiable risk factors during development that impact adult health and welfare (219). The research will investigate relationships between genotype, environment, and health and behavior outcomes at different life stages. There are also breed-specific longitudinal cohort studies such as the Golden Retriever Lifetime Study run by the Morris Animal Foundation in the USA, which is collecting data on the lifestyle, environment, behavior and health of 3,000 dogs recruited between 2012 and 2015, including annual biological samples (66, 220, 221). The ongoing Dogslife epidemiological project (University of Edinburgh, University of Manchester, University of Liverpool and the Kennel Club) recruits UK pedigree Labrador Retrievers born after January 2010 (*n* = 6,084 dogs by December 2015) (222, 223). Owners complete questionnaires each month for the first year of their dog's life and every 3 months thereafter (222, 223). In the USA, the ongoing Dog Aging Project (University of Washington and Texas A&M University) has recruited tens of thousands of companion dogs to explore aspects of “health-span” i.e., the period of life spent free from disease (224). The Norwegian School of Veterinary Science cohort established to investigate skeletal disease in four large dog breeds (*n* = 700 puppies recruited 1998–2001), is notable as an example of a longitudinal cohort in which dog litters were recruited from the time of the dam's mating, and data were obtained from breeders, owners and veterinarians (225, 226).

Longitudinal cat registries and cohort studies appear to be scarce. The landmark Bristol Cats Study led by the University of Bristol is the first reported birth cohort study of kittens (227). Cats were registered between the ages of 8 and 16 weeks, and owners complete questionnaires at set intervals. Analyses reported to date include the prevalence of and risk factors for obesity, and owner-reported lower urinary tract signs (228–230). The Cat Phenotype and Health Information Registry in the USA (UC Davis Veterinary Medicine) collects DNA samples from healthy and diseased cats with long-term follow-up where possible (231).

Data on epigenetic mechanisms linking early-life experiences to adult disorders in dogs and cats are scant; they are needed to help confirm and understand developmental programming, and unravel the effects of genetic background. Methods for profiling genome-wide DNA methylation are well established, and much can be achieved before attempting to identify the specific genes responsible for an epigenetically determined phenotype (232, 233). Epigenetic mechanisms other than DNA methylation should also be studied, such as histone modifications, including but not limited to methylation and acetylation, and non-coding RNA that can regulate gene expression during cell differentiation and development (234). A publicly available repository of canine epigenomic data (BarkBase) has recently been established, comprising the results of RNA sequencing and assays determining chromatin accessibility across the genome (235). The database includes 27 different adult tissues and five fetal tissue types at four embryonic timepoints. The Royal Veterinary College has instituted the Companion Animal Brain Bank—a standardized collection of brain tissue and other biological samples from dogs and cats euthanized with neurological conditions, together with appropriate controls. Although such tissue banks could be used to investigate changes in the epigenome, including those associated with disease, without corresponding data on environmental aspects of pregnancy and early life, they will not provide evidence of developmental programming.

The effect of early-life experiences and developmental programming on at least some physiological characteristics will be affected by genotypic differences between the many breeds and mixed breeds. Targeting research to specific breeds on the basis of their propensity for developing the disease or behavior of interest can be advantageous, e.g., the Labrador Retriever for obesity.

There are fewer breeds of cats to contend with in DOHaD research, but overall the knowledge gaps are greater than in dogs. There appear to be fewer longitudinal field data in cats compared with dogs, and there is probably less public awareness of the potential impact of developmental programming on chronic diseases.

## Concluding remarks

There is direct evidence for early-life risk factors associated with obesity and behavioral problems in dogs and cats, and to a much lesser extent CE in dogs. However, multidisciplinary prospective long-term research is needed to confirm DOHaD in these species. Extensive data from other species provide a scientific foundation to help prioritize early-life events and exposures for investigation. The diversity of dog and cat breeds, breeding management and lifestyles adds complexity to such research. It is believed that breeders, owners and veterinary surgeons each have a critical window of opportunity in one or more of the life stages from preconception to the end of the dog or cat's growth phase in which to promote programming beneficial to long-term health. An appreciation by each of these groups of the overall window of development may also help to foster shared responsibility, transparency and information sharing.

Dogs and cats are considered to be family members, and yet veterinary medicine struggles to treat common conditions that adversely impact pets' quality of life, the special owner–pet bond, and the health benefits pets can bring to individuals and society. Preventive medicine and husbandry practices from preconception onwards must take a higher priority and be fueled by a better understanding of developmental programming at the population level.

## Author contributions

VG, SC, GE, OF, AG, JS, CV, PC-P, and FP contributed substantially to the conception of the article and to interpretation of data presented. All authors critical reviewed the manuscript for important intellectual content. The authors take full responsibility for the scientific content of the paper and they have all approved the submitted version.

## References

[1] Mila H Grellet A Feugier A Chastant-Maillard S. Differential impact of birth weight and early growth on neonatal mortality in puppies. J Anim Sci. (2015) 93:4436–42. 10.2527/jas.2015-8971 26440343

[2] Mugnier A Chastant-Maillard S Mila H Lyazrhi F Guiraud F Adib-Lesaux A. Low and very low birth weight in puppies: definitions, risk factors and survival in a large-scale population. BMC Vet Res. (2020) 16:354. 10.1186/s12917-020-02577-z 32972422 PMC7517789

[3] Mugnier A Mila H Guiraud F Brévaux J Lecarpentier M Martinez C. Birth weight as a risk factor for neonatal mortality: breed-specific approach to identify at-risk puppies. Prev Vet Med. (2019) 171:104746. 10.1016/j.prevetmed.2019.104746 31491708

[4] Leclerc L Thorin C Flanagan J Biourge V Serisier S Nguyen P. Higher neonatal growth rate and body condition score at 7 months are predictive factors of obesity in adult female Beagle dogs. BMC Vet Res. (2017) 13:104. 10.1186/s12917-017-0994-7 28407742 PMC5390368

[5] Mugnier A Morin A Cellard F Devaux L Delmas M Adib-Lesaux A. Association between birth weight and risk of overweight at adulthood in Labrador dogs. PLoS ONE. (2020) 15:e0243820. 10.1371/journal.pone.0243820 33301504 PMC7728192

[6] Linnér A Almgren M. Epigenetic programming-the important first 1000 days. Acta Paediatr. (2020) 109:443–52. 10.1111/apa.15050 31603247

[7] Bianco-Miotto T Craig JM Gasser YP van Dijk SJ Ozanne SE. Epigenetics and DOHaD: from basics to birth and beyond. J Dev Orig Health Dis. (2017) 8:513–9. 10.1017/S2040174417000733 28889823

[8] Chavatte-Palmer P Tarrade A Rousseau-Ralliard D. Diet before and during pregnancy and offspring health: the importance of animal models and what can be learned from them. Int J Environ Res Public Health. (2016) 13:586. 10.3390/ijerph13060586 27314367 PMC4924043

[9] Triantaphyllopoulos KA Ikonomopoulos I Bannister AJ. Epigenetics and inheritance of phenotype variation in livestock. Epigenetics Chromatin. (2016) 9:31. 10.1186/s13072-016-0081-5 27446239 PMC4955263

[10] German AJ Woods GRT Holden SL Brennan L Burke C. Dangerous trends in pet obesity. Vet Rec. (2018) 182:25. 10.1136/vr.k2 29305476 PMC5806590

[11] Salonen M Sulkama S Mikkola S Puurunen J Hakanen E Tiira K. Prevalence, comorbidity, and breed differences in canine anxiety in 13,700 Finnish pet dogs. Sci Rep. (2020) 10:2962. 10.1038/s41598-020-59837-z 32139728 PMC7058607

[12] Dandrieux JRS Mansfield CS. Chronic enteropathy in canines: prevalence, impact and management strategies. Vet Med (Auckl). (2019) 10:203–14. 10.2147/VMRR.S162774 31828025 PMC6902862

[13] Eagan BH Gordon E Protopopova A. Reasons for guardian-relinquishment of dogs to shelters: animal and regional predictors in British Columbia, Canada. Front Vet Sci. (2022) 9:857634. 10.3389/fvets.2022.857634 35498734 PMC9050194

[14] Salman MD Hutchison J Ruch-Gallie R Kogan L New JC Jr Kass PH. Behavioral reasons for relinquishment of dogs and cats to 12 shelters. J Appl Anim Welf Sci. (2000) 3:93–106. 10.1207/S15327604JAWS0302_2

[15] Serpell J Duffy DL Jagoe A. Becoming a dog: early experience and the development of behavior. In:Serpell J, editor. The Domestic Dog: Its Evolution Behavior and Interactions with People. 2nd edition. Cambridge: Cambridge University Press (2016). p. 94–117. 10.1017/9781139161800.006

[16] Gee NR Rodriguez KE Fine AH Trammell JP. Dogs supporting human health and well-being: a biopsychosocial approach. Front Vet Sci. (2021) 8:630465. 10.3389/fvets.2021.630465 33860004 PMC8042315

[17] Qureshi AI Memon MZ Vazquez G Suri MF. Cat ownership and the risk of fatal cardiovascular diseases. Results from the second national health and nutrition examination study mortality follow-up study. J Vasc Interv Neurol. (2009) 2:132–5. 22518240 PMC3317329

[18] FEDIAF. Facts and Figures 2021 European Overview (2022). Available online at: https://europeanpetfood.org/about/annual-report/ (accessed May 9, 2022).

[19] AVMA. Pet Ownership and Demographics Sourcebook 2022. Washington, DC: American Veterinary Medical Association (2022).

[20] The American Pet Products Association. Pet Industry Market Size, Trends and Ownership Statistics 2021 (2022). Available online at: https://www.americanpetproducts.org/press_industrytrends.asp (accessed July 11, 2022).

[21] Federation, Cynologique International. Presentation of Our Organisation. Available online at: http://www.fci.be/en/Presentation-of-our-organisation-4.html (accessed August 4, 2021).

[22] Grandjean D Haymann F Andre C Bacque H Bedossa T Boogaets C. The Dog Encyclopaedia. Third ed. France: Royal Canin SAS (2021).p. 930.

[23] Hawthorne AJ Booles D Nugent PA Gettinby G Wilkinson J. Body-weight changes during growth in puppies of different breeds. J Nutr. (2004) 134:2027s−30s. 10.1093/jn/134.8.2027S 15284394

[24] The Cat Fanciers' Association. CFA Breeds. Available online at: https://cfa.org/breeds/ (accessed August 4, 2021).

[25] Caney SA Fontbonne A Deputte BL German AJ Dethioux F German AJ. Royal Canin. The Cat Encyclopedia. Second ed. Havasupai, Tewa: Aniwa Publishing (2017). p. 496.

[26] Pieri N Souza AF Casals JB Roballo K Ambrósio CE Martins DS. Comparative development of embryonic age by organogenesis in domestic dogs and cats. Reprod Domest Anim. (2015) 50:625–31. 10.1111/rda.12539 25990819

[27] Binder C Aurich C Reifinger M Aurich J. Spontaneous ovulation in cats-uterine findings and correlations with animal weight and age. Anim Reprod Sci. (2019) 209:106167. 10.1016/j.anireprosci.2019.106167 31514917

[28] Reynaud K Saint-Dizier M Fontbonne A Thoumire S Chastant-Maillard S. Follicle growth, oocyte maturation, embryo development, and reproductive biotechnologies in dog and cat. Clin Theriogenology. (2020) 12:189–202.

[29] Reynaud K Fontbonne A Marseloo N Thoumire S Chebrout M de Lesegno CV. In vivo meiotic resumption, fertilization and early embryonic development in the bitch. Reproduction. (2005) 130:193–201. 10.1530/rep.1.00500 16049157

[30] Kopelman PG. Obesity as a medical problem. Nature. (2000) 404:635–43. 10.1038/35007508 10766250

[31] German AJ. The growing problem of obesity in dogs and cats. J Nutr. (2006) 136:1940s−6s. 10.1093/jn/136.7.1940S 16772464

[32] Ward E German AJ Churchhill JA. Association for Pet Obesity Prevention. The Global Pet Obesity Initiative Position Statement (2019). Available online at: https://petobesityprevention.org/about/#GPOI (accessed May 9, 2022).

[33] Verbrugghe A. Epidemiology of small animal obesity. In:Cline MG Murphy M, editors. Obesity in the Dog and Cat. Boca Raton, FL: CRC Press (2019). p. 1–13. 10.1201/9781315151625-1

[34] Bjornvad CR Nielsen DH Armstrong PJ McEvoy F Hoelmkjaer KM Jensen KS. Evaluation of a nine-point body condition scoring system in physically inactive pet cats. Am J Vet Res. (2011) 72:433–7. 10.2460/ajvr.72.4.433 21453142

[35] Laflamme D. Development and validation of a body condition score system for cats: a clinical tool. Feline Pract. (1997) 25:13–8.

[36] Association for Pet Obesity Prevention,. 2018 Pet Obesity Survey Results (2019). Available from: https://petobesityprevention.org/2018 (accessed October 19, 2021).

[37] Tropf M Nelson OL Lee PM Weng HY. Cardiac and metabolic variables in obese dogs. J Vet Intern Med. (2017) 31:1000–7. 10.1111/jvim.14775 28608635 PMC5508341

[38] Bach JF Rozanski EA Bedenice D Chan DL Freeman LM Lofgren JL. Association of expiratory airway dysfunction with marked obesity in healthy adult dogs. Am J Vet Res. (2007) 68:670–5. 10.2460/ajvr.68.6.670 17542702

[39] Manens J Ricci R Damoiseaux C Gault S Contiero B Diez M. Effect of body weight loss on cardiopulmonary function assessed by 6-minute walk test and arterial blood gas analysis in obese dogs. J Vet Intern Med. (2014) 28:371–8. 10.1111/jvim.12260 24351032 PMC4858022

[40] Henegar JR Bigler SA Henegar LK Tyagi SC Hall JE. Functional and structural changes in the kidney in the early stages of obesity. J Am Soc Nephrol. (2001) 12:1211–7. 10.1681/ASN.V1261211 11373344

[41] Tvarijonaviciute A Ceron JJ Holden SL Biourge V Morris PJ German AJ. Effect of weight loss in obese dogs on indicators of renal function or disease. J Vet Intern Med. (2013) 27:31–8. 10.1111/jvim.12029 23278113

[42] German AJ Hervera M Hunter L Holden SL Morris PJ Biourge V. Improvement in insulin resistance and reduction in plasma inflammatory adipokines after weight loss in obese dogs. Domest Anim Endocrinol. (2009) 37:214–26. 10.1016/j.domaniend.2009.07.001 19674864

[43] Tvarijonaviciute A Ceron JJ Holden SL Cuthbertson DJ Biourge V Morris PJ. Obesity-related metabolic dysfunction in dogs: a comparison with human metabolic syndrome. BMC Vet Res. (2012) 8:147. 10.1186/1746-6148-8-147 22929809 PMC3514388

[44] Salt C Morris PJ Wilson D Lund EM German AJ. Association between life span and body condition in neutered client-owned dogs. J Vet Intern Med. (2019) 33:89–99. 10.1111/jvim.15367 30548336 PMC6335446

[45] German AJ Holden SL Wiseman-Orr ML Reid J Nolan AM Biourge V. Quality of life is reduced in obese dogs but improves after successful weight loss. Vet J. (2012) 192:428–34. 10.1016/j.tvjl.2011.09.015 22075257

[46] Lund EM Armstrong PJ Kirk CA Klausner JS. Prevalence and risk factors for obesity in adult dogs from private US veterinary practices. Intern J Appl Res Vet Med. (2006) 4:177.

[47] Perez Alenza MD Peña L del Castillo N Nieto AI. Factors influencing the incidence and prognosis of canine mammary tumours. J Small Anim Pract. (2000) 41:287–91. 10.1111/j.1748-5827.2000.tb03203.x 10976622

[48] Brown DC Conzemius M Shofer F. Body weight as a predisposing factor for humeral condylar fractures, cranial cruciate rupture and intervertebral disc disease in Cocker Spaniels. Vet Comp Orthop Traumatol. (1996) 9:75–8. 10.1055/s-0038-1632506

[49] van Hagen MA Ducro BJ van den Broek J Knol BW. Incidence, risk factors, and heritability estimates of hind limb lameness caused by hip dysplasia in a birth cohort of boxers. Am J Vet Res. (2005) 66:307–12. 10.2460/ajvr.2005.66.307 15757132

[50] Gates MC Zito S Harvey LC Dale A Walker JK. Assessing obesity in adult dogs and cats presenting for routine vaccination appointments in the north island of New Zealand using electronic medical records data. N Z Vet J. (2019) 67:126–33. 10.1080/00480169.2019.1585990 30806171

[51] Parker VJ Orcutt E Love L. Pathophysiology of obesity: comorbidities and anesthetic considerations. In:Cline MG Murphy M, editors. Obesity in the Dog and Cat. First ed. Boca Raton: CRC Press (2019). p. 40–61. 10.1201/9781315151625-3

[52] Chiang CF Villaverde C Chang WC Fascetti AJ Larsen JA. Prevalence, risk factors, and disease associations of overweight and obesity in cats that visited the veterinary medical teaching hospital at the University of California, Davis from January 2006 to December 2015. Top Companion Anim Med. (2022) 47:100620. 10.1016/j.tcam.2021.100620 34936906

[53] Teng KT McGreevy PD Toribio J Raubenheimer D Kendall K Dhand NK. Associations of body condition score with health conditions related to overweight and obesity in cats. J Small Anim Pract. (2018) 59:603–15. 10.1111/jsap.12905 30033652

[54] Teng KT McGreevy PD Toribio JL Raubenheimer D Kendall K Dhand NK. Strong associations of nine-point body condition scoring with survival and lifespan in cats. J Feline Med Surg. (2018) 20:1110–8. 10.1177/1098612X17752198 29393723 PMC11104206

[55] Flanagan J Bissot T Hours MA Moreno B German AJ. An international multi-centre cohort study of weight loss in overweight cats: differences in outcome in different geographical locations. PLoS ONE. (2018) 13:e0200414. 10.1371/journal.pone.0200414 30044843 PMC6059437

[56] Feldhahn J Rand J Martin G. Insulin sensitivity in normal and diabetic cats. J Feline Med Surg. (1999) 1:107–15. 10.1016/S1098-612X(99)90067-0 11919024 PMC10822474

[57] Lund EM Armstrong PJ Kirk CA Klausmer JS. Prevalence and risk factors for obesity in adult cats from private US veterinary practices. Intern J Appl Res Vet Med. (2005) 3:88–96.

[58] Valtolina C Favier RP. Feline hepatic lipidosis. Vet Clin North Am Small Anim Pract. (2017) 47:683–702. 10.1016/j.cvsm.2016.11.014 28108035

[59] Salt C Morris PJ Butterwick RF Lund EM Cole TJ German AJ. Comparison of growth patterns in healthy dogs and dogs in abnormal body condition using growth standards. PLoS ONE. (2020) 15:e0238521. 10.1371/journal.pone.0238521 32966286 PMC7510995

[60] Bjørnvad CR Gloor S Johansen SS Sandøe P Lund TB. Neutering increases the risk of obesity in male dogs but not in bitches—a cross-sectional study of dog- and owner-related risk factors for obesity in Danish companion dogs. Prev Vet Med. (2019) 170:104730. 10.1016/j.prevetmed.2019.104730 31421500

[61] Colliard L Ancel J Benet JJ Paragon BM Blanchard G. Risk factors for obesity in dogs in France. J Nutr. (2006) 136:1951s−4s. 10.1093/jn/136.7.1951S 16772466

[62] German AJ Blackwell E Evans M Westgarth C. Overweight dogs exercise less frequently and for shorter periods: results of a large online survey of dog owners from the UK. J Nutr Sci. (2017) 6:e11. 10.1017/jns.2017.6 28620486 PMC5465938

[63] McGreevy PD Thomson PC Pride C Fawcett A Grassi T Jones B. Prevalence of obesity in dogs examined by Australian veterinary practices and the risk factors involved. Vet Rec. (2005) 156:695–702. 10.1136/vr.156.22.695 15923551

[64] Perry LM Shmalberg J Tanprasertsuk J Massey D Honaker RW Jha AR. Risk factors associated with canine overweightness and obesity in an owner-reported survey. bioRxiv. (2020). 10.1101/2020.01.06.896399

[65] Vendramini THA Amaral AR Pedrinelli V Zafalon RVA Rodrigues RBA Brunetto MA. Neutering in dogs and cats: current scientific evidence and importance of adequate nutritional management. Nutr Res Rev. (2020) 33:134–44. 10.1017/S0954422419000271 31931899

[66] Simpson M Albright S Wolfe B Searfoss E Street K Diehl K. Age at gonadectomy and risk of overweight/obesity and orthopedic injury in a cohort of Golden Retrievers. PLoS ONE. (2019) 14:e0209131. 10.1371/journal.pone.0209131 31314808 PMC6636707

[67] Lefebvre SL Yang M Wang M Elliott DA Buff PR Lund EM. Effect of age at gonadectomy on the probability of dogs becoming overweight. J Am Vet Med Assoc. (2013) 243:236–43. 10.2460/javma.243.2.236 23822081

[68] Salt C Morris PJ German AJ Wilson D Lund EM Cole TJ. Growth standard charts for monitoring bodyweight in dogs of different sizes. PLoS ONE. (2017) 12:e0182064. 10.1371/journal.pone.0182064 28873413 PMC5584974

[69] Bermingham EN Thomas DG Cave NJ Morris PJ Butterwick RF German AJ. Energy requirements of adult dogs: a meta-analysis. PLoS ONE. (2014) 9:e109681. 10.1371/journal.pone.0109681 25313818 PMC4196927

[70] Hoenig M Ferguson DC. Effects of neutering on hormonal concentrations and energy requirements in male and female cats. Am J Vet Res. (2002) 63:634–9. 10.2460/ajvr.2002.63.634 12013460

[71] Kutzler MA. Possible relationship between long-term adverse health effects of gonad-removing surgical sterilization and luteinizing hormone in dogs. Animals (Basel). (2020) 10:599. 10.3390/ani10040599 32244716 PMC7222805

[72] Mugnier A Cellard F Morin A Guiraud F Mariani C Adib-Lesaux A. Impact of neonatal and adult factors on body condition of Labrador dogs. Reprod Domest Anim. (2019) 54:36–7.

[73] Dewey KG Güngör D Donovan SM Madan EM Venkatramanan S Davis TA. Breastfeeding and risk of overweight in childhood and beyond: a systematic review with emphasis on sibling-pair and intervention studies. Am J Clin Nutr. (2021) 114:1774–90. 10.1093/ajcn/nqab206 34224561 PMC8830309

[74] Palou M Picó C Palou A. Leptin as a breast milk component for the prevention of obesity. Nutr Rev. (2018) 76:875–92. 10.1093/nutrit/nuy046 30285146

[75] van Lent D Vernooij JCM Corbee RJ. Kittens that nurse 7 weeks or longer are less likely to become overweight adult cats. Animals. (2021) 11:3434. 10.3390/ani11123434 34944211 PMC8697871

[76] Serisier S Feugier A Venet C Biourge V German AJ. Faster growth rate in ad libitum-fed cats: a risk factor predicting the likelihood of becoming overweight during adulthood. J Nutr Sci. (2013) 2:e11. 10.1017/jns.2013.10 25191559 PMC4153074

[77] Cave NJ Bridges JP Weidgraaf K Thomas DG. Nonlinear mixed models of growth curves from domestic shorthair cats in a breeding colony, housed in a seasonal facility to predict obesity. J Anim Physiol Anim Nutr (Berl). (2018) 102:1390–400. 10.1111/jpn.12930 29932481

[78] Opsomer H Liesegang A Brugger D Wichert B. Growth curves and body condition of young cats and their relation to maternal body condition. Animals. (2022) 12:1373. 10.3390/ani12111373 35681836 PMC9179872

[79] Fettman MJ Stanton CA Banks LL Hamar DW Johnson DE Hegstad RL. Effects of neutering on bodyweight, metabolic rate and glucose tolerance of domestic cats. Res Vet Sci. (1997) 62:131–6. 10.1016/S0034-5288(97)90134-X 9243711

[80] Wall M Cave NJ Vallee E. Owner and cat-related risk factors for feline overweight or obesity. Front Vet Sci. (2019) 6:266. 10.3389/fvets.2019.00266 31482097 PMC6709657

[81] Alexander LG Salt C Thomas G Butterwick R. Effects of neutering on food intake, body weight and body composition in growing female kittens. Br J Nutr. (2011) 106 Suppl 1:S19–23. 10.1017/S0007114511001851 22005425

[82] Kanchuk ML Backus RC Calvert CC Morris JG Rogers QR. Weight gain in gonadectomized normal and lipoprotein lipase-deficient male domestic cats results from increased food intake and not decreased energy expenditure. J Nutr. (2003) 133:1866–74. 10.1093/jn/133.6.1866 12771331

[83] Wei A Fascetti AJ Kim K Lee A Graham JL Havel PJ. Early effects of neutering on energy expenditure in adult male cats. PLoS ONE. (2014) 9:e89557. 10.1371/journal.pone.0089557 24586869 PMC3935885

[84] Bermingham EN Thomas DG Morris PJ Hawthorne AJ. Energy requirements of adult cats. Br J Nutr. (2010) 103:1083–93. 10.1017/S000711450999290X 20100376

[85] Allaway D Gilham M Colyer A Morris PJ. The impact of time of neutering on weight gain and energy intake in female kittens. J Nutr Sci. (2017) 6:e19. 10.1017/jns.2017.20 28630696 PMC5468748

[86] de Godoy MRC and Pancosma Comparative Gut Physiology Symposium. All about appetite regulation: effects of diet and gonadal steroids on appetite regulation and food intake of companion animals. J Anim Sci. (2018) 96:3526–36. 10.1093/jas/sky146 29982536 PMC6095297

[87] Marousez L Lesage J Eberlé D. Epigenetics: linking early postnatal nutrition to obesity programming? Nutrients. (2019) 11:2966. 10.3390/nu11122966 31817318 PMC6950532

[88] Moullé VS Parnet P. Effects of nutrient intake during pregnancy and lactation on the endocrine pancreas of the offspring. Nutrients. (2019) 11:2708. 10.3390/nu11112708 31717308 PMC6893668

[89] van Dijk SJ Tellam RL Morrison JL Muhlhausler BS Molloy PL. Recent developments on the role of epigenetics in obesity and metabolic disease. Clin Epigenetics. (2015) 7:66. 10.1186/s13148-015-0101-5 27408648 PMC4940755

[90] Picó C Reis F Egas C Mathias P Matafome P. Lactation as a programming window for metabolic syndrome. Eur J Clin Invest. (2021) 51:e13482. 10.1111/eci.13482 33350459

[91] Ouni M Schürmann A. Epigenetic contribution to obesity. Mammalian Genome. (2020) 31:134–45. 10.1007/s00335-020-09835-3 32279091 PMC7368865

[92] Hoffman DJ Powell TL Barrett ES Hardy DB. Developmental origins of metabolic diseases. Physiol Rev. (2021) 101:739–95. 10.1152/physrev.00002.2020 33270534 PMC8526339

[93] Hoffman DJ Reynolds RM Hardy DB. Developmental origins of health and disease: current knowledge and potential mechanisms. Nutr Rev. (2017) 75:951–70. 10.1093/nutrit/nux053 29186623

[94] Kaspar D Hastreiter S Irmler M Hrabé de Angelis M Beckers J. Nutrition and its role in epigenetic inheritance of obesity and diabetes across generations. Mamm Genome. (2020) 31:119–33. 10.1007/s00335-020-09839-z 32350605 PMC7368866

[95] Obri A Serra D Herrero L Mera P. The role of epigenetics in the development of obesity. Biochem Pharmacol. (2020) 177:113973. 10.1016/j.bcp.2020.113973 32283053

[96] Bouret SG Draper SJ Simerly RB. Trophic action of leptin on hypothalamic neurons that regulate feeding. Science. (2004) 304:108–10. 10.1126/science.1095004 15064420

[97] Breton C. The hypothalamus-adipose axis is a key target of developmental programming by maternal nutritional manipulation. J Endocrinol. (2013) 216:R19–31. 10.1530/JOE-12-0157 23108716

[98] Hur SSJ Cropley JE Suter CM. Paternal epigenetic programming: evolving metabolic disease risk. J Mol Endocrinol. (2017) 58:R159–r68. 10.1530/JME-16-0236 28100703

[99] Hullinger RL. A histomorphological study of age changes in the canine adrenal gland. Retrospective Theses and Dissertations. Ames, IA: Iowa State University of Science and Technology (1966).

[100] Buddington RK Elnif J Malo C Donahoo JB. Activities of gastric, pancreatic, and intestinal brush-border membrane enzymes during postnatal development of dogs. Am J Vet Res. (2003) 64:627–34. 10.2460/ajvr.2003.64.627 12755304

[101] Day MJ. Immune system development in the dog and cat. J Comp Pathol. (2007) 137 Suppl 1:S10–5. 10.1016/j.jcpa.2007.04.005 17560591

[102] Fox MW. Overview and critique of stages and periods in canine development. Dev Psychobiol. (1971) 4:37–54. 10.1002/dev.420040104 4950117

[103] Jeusette I Daminet S Nguyen P Shibata H Saito M Honjoh T. Effect of ovariectomy and ad libitum feeding on body composition, thyroid status, ghrelin and leptin plasma concentrations in female dogs. J Anim Physiol Anim Nutr. (2006) 90:12–8. 10.1111/j.1439-0396.2005.00612.x 16422764

[104] Spray CM Widdowson EM. The effect of growth and development on the composition of mammals. Br J Nutr. (1950) 4:332–53. 10.1079/BJN19500058 14812082

[105] Sheng HP Huggins RA. Changes in water, protein, sodium, potassium, and chloride in tissues with growth of the Beagle. Growth. (1975) 39:137–57. 1132771

[106] Wang W Brooks M Gardner C Milgram N. Effect of neuroactive nutritional supplementation on body weight and composition in growing puppies. J Nutr Sci. (2017) 6:e56. 10.1017/jns.2017.57 29209495 PMC5705811

[107] Salt C German AJ Henzel KS Butterwick RF. Growth standard charts for monitoring bodyweight in intact domestic shorthair kittens from the USA. PLoS ONE. (2022) 17:e0277531. 10.1371/journal.pone.0277531 36409712 PMC9678321

[108] Fischer MM Kessler AM Kieffer DA Knotts TA Kim K Wei A. Effects of obesity, energy restriction and neutering on the faecal microbiota of cats. Br J Nutr. (2017) 118:513–24. 10.1017/S0007114517002379 28958218

[109] Forster GM Stockman J Noyes N Heuberger AL Broeckling CD Bantle CM. A comparative study of serum biochemistry, metabolome and microbiome parameters of clinically healthy, normal weight, overweight, and obese companion dogs. Top Companion Anim Med. (2018) 33:126–35. 10.1053/j.tcam.2018.08.003 30502863

[110] Handl S German AJ Holden SL Dowd SE Steiner JM Heilmann RM. Faecal microbiota in lean and obese dogs. FEMS Microbiol Ecol. (2013) 84:332–43. 10.1111/1574-6941.12067 23301868

[111] Park HJ Lee SE Kim HB Isaacson RE Seo KW Song KH. Association of obesity with serum leptin, adiponectin, and serotonin and gut microflora in Beagle dogs. J Vet Intern Med. (2015) 29:43–50. 10.1111/jvim.12455 25407880 PMC4858068

[112] Kieler IN Mølbak L Hansen LL Hermann-Bank ML Bjornvad CR. Overweight and the feline gut microbiome—a pilot study. J Anim Physiol Anim Nutr. (2016) 100:478–84. 10.1111/jpn.12409 26452635

[113] Macedo HT Rentas MF Vendramini THA Macegoza MV Amaral AR Jeremias JT. Weight-loss in obese dogs promotes important shifts in fecal microbiota profile to the extent of resembling microbiota of lean dogs. Anim Microbiome. (2022) 4:6. 10.1186/s42523-021-00160-x 34991726 PMC8740440

[114] Phungviwatnikul T Lee AH Belchik SE Suchodolski JS Swanson KS. Weight loss and high-protein, high-fiber diet consumption impact blood metabolite profiles, body composition, voluntary physical activity, fecal microbiota, and fecal metabolites of adult dogs. J Anim Sci. (2022) 100:skab379. 10.1093/jas/skab379 34967874 PMC8846339

[115] Söder J Wernersson S Höglund K Hagman R Lindåse S Dicksved J. Composition and short-term stability of gut microbiota in lean and spontaneously overweight healthy Labrador Retriever dogs. Acta Vet Scand. (2022) 64:8. 10.1186/s13028-022-00628-z 35346308 PMC8962211

[116] Tal M Weese JS Gomez DE Hesta M Steiner JM Verbrugghe A. Bacterial fecal microbiota is only minimally affected by a standardized weight loss plan in obese cats. BMC Vet Res. (2020) 16:112. 10.1186/s12917-020-02318-2 32293441 PMC7161297

[117] Bermingham EN Kittelmann S Young W Kerr KR Swanson KS Roy NC. Post-weaning diet affects faecal microbial composition but not selected adipose gene expression in the cat (*Felis catus*). PLoS ONE. (2013) 8:e80992. 10.1371/journal.pone.0080992 24312255 PMC3842929

[118] Pocheron AL Le Dréan G Billard H Moyon T Pagniez A Heberden C. Maternal microbiota transfer programs offspring eating behavior. Front Microbiol. (2021) 12:672224. 10.3389/fmicb.2021.672224 34211445 PMC8239415

[119] Cuevas-Sierra A Ramos-Lopez O Riezu-Boj JI Milagro FI Martinez JA. Diet, gut microbiota, and obesity: links with host genetics and epigenetics and potential applications. Adv Nutr. (2019) 10(suppl_1):s17–s30. 10.1093/advances/nmy078 30721960 PMC6363528

[120] Krautkramer KA Kreznar JH Romano KA Vivas EI Barrett-Wilt GA Rabaglia ME. Diet-microbiota interactions mediate global epigenetic programming in multiple host tissues. Mol Cell. (2016) 64:982–92. 10.1016/j.molcel.2016.10.025 27889451 PMC5227652

[121] Shock T Badang L Ferguson B Martinez-Guryn K. The interplay between diet, gut microbes, and host epigenetics in health and disease. J Nutr Biochem. (2021) 95:108631. 10.1016/j.jnutbio.2021.108631 33789148 PMC8355029

[122] Woo V Alenghat T. Epigenetic regulation by gut microbiota. Gut Microbes. (2022) 14:2022407. 10.1080/19490976.2021.2022407 35000562 PMC8744890

[123] Sharma M Li Y Stoll ML Tollefsbol TO. The epigenetic connection between the gut microbiome in obesity and diabetes. Front Genet. (2019) 10:1329. 10.3389/fgene.2019.01329 32010189 PMC6974692

[124] Tramper-Stranders G Ambrozej D Arcolaci A Atanaskovic-Markovic M Boccabella C Bonini M. Dangerous liaisons: bacteria, antimicrobial therapies, and allergic diseases. Allergy. (2021) 76:3276–91. 10.1111/all.15046 34390006

[125] Loos RJF Yeo GSH. The genetics of obesity: from discovery to biology. Nat Rev Genet. (2022) 23:120–33. 10.1038/s41576-021-00414-z 34556834 PMC8459824

[126] Pegram C Raffan E White E Ashworth AH Brodbelt DC Church DB. Frequency, breed predisposition and demographic risk factors for overweight status in dogs in the UK. J Small Anim Pract. (2021) 62:521–30. 10.1111/jsap.13325 33754373

[127] Wallis N Raffan E. The genetic basis of obesity and related metabolic diseases in humans and companion animals. Genes. (2020) 11:1378. 10.3390/genes11111378 33233816 PMC7699880

[128] Mankowska M Krzeminska P Graczyk M Switonski M. Confirmation that a deletion in the POMC gene is associated with body weight of Labrador Retriever dogs. Res Vet Sci. (2017) 112:116–8. 10.1016/j.rvsc.2017.02.014 28235700

[129] Raffan E Dennis RJ O'Donovan CJ Becker JM Scott RA Smith SP. A deletion in the canine POMC gene is associated with weight and appetite in obesity-prone Labrador Retriever dogs. Cell Metab. (2016) 23:893–900. 10.1016/j.cmet.2016.04.012 27157046 PMC4873617

[130] Moore PF Woo JC Vernau W Kosten S Graham PS. Characterization of feline T cell receptor gamma (TCRG) variable region genes for the molecular diagnosis of feline intestinal T cell lymphoma. Vet Immunol Immunopathol. (2005) 106:167–78. 10.1016/j.vetimm.2005.02.014 15963816

[131] Marsilio S. Feline chronic enteropathy. J Small Anim Pract. (2021) 62:409–19. 10.1111/jsap.13332 33821508

[132] Kilian E Suchodolski JS Hartmann K Mueller RS Wess G Unterer S. Long-term effects of canine parvovirus infection in dogs. PLoS ONE. (2018) 13:e0192198. 10.1371/journal.pone.0192198 29547647 PMC5856261

[133] Sato-Takada K Flemming AM Voordouw MJ Carr AP. Parvovirus enteritis and other risk factors associated with persistent gastrointestinal signs in dogs later in life: a retrospective cohort study. BMC Vet Res. (2022) 18:96. 10.1186/s12917-022-03187-7 35277172 PMC8915519

[134] Hemida M Vuori KA Moore R Anturaniemi J Hielm-Björkman A. Early life modifiable exposures and their association with owner reported inflammatory bowel disease symptoms in adult dogs. Front Vet Sci. (2021) 8:552350. 10.3389/fvets.2021.552350 33598486 PMC7882719

[135] Sundburg CR Belanger JM Bannasch DL Famula TR Oberbauer AM. Gonadectomy effects on the risk of immune disorders in the dog: a retrospective study. BMC Vet Res. (2016) 12:278. 10.1186/s12917-016-0911-5 27931211 PMC5146839

[136] Jergens AE. Feline idiopathic inflammatory bowel disease: what we know and what remains to be unraveled. J Feline Med Surg. (2012) 14:445–58. 10.1177/1098612X12451548 22736679 PMC10822384

[137] Fofanova TY Petrosino JF Kellermayer R. Microbiome-epigenome interactions and the environmental origins of inflammatory bowel diseases. J Pediatr Gastroenterol Nutr. (2016) 62:208–19. 10.1097/MPG.0000000000000950 26308318 PMC4724338

[138] Indrio F Martini S Francavilla R Corvaglia L Cristofori F Mastrolia SA. Epigenetic matters: the link between early nutrition, microbiome, and long-term health development. Front Pediatr. (2017) 5:178. 10.3389/fped.2017.00178 28879172 PMC5572264

[139] Pilla R Suchodolski JS. The role of the canine gut microbiome and metabolome in health and gastrointestinal disease. Front Vet Sci. (2019) 6:498. 10.3389/fvets.2019.00498 31993446 PMC6971114

[140] Garrigues Q Apper E Chastant S Mila H. Gut microbiota development in the growing dog: a dynamic process influenced by maternal, environmental and host factors. Front Vet Sci. (2022) 9:964649. 10.3389/fvets.2022.964649 36118341 PMC9478664

[141] Deusch O O'Flynn C Colyer A Morris P Allaway D Jones PG. Deep illumina-based shotgun sequencing reveals dietary effects on the structure and function of the fecal microbiome of growing kittens. PLoS ONE. (2014) 9:e101021. 10.1371/journal.pone.0101021 25010839 PMC4091873

[142] Sung C-H Marsilio S Chow B Zornow KA Slovak JE Pilla R. A dysbiosis index to evaluate the fecal microbiota in healthy cats and cats with chronic enteropathies. J Feline Med Surg. (2022) 24:e1–12. 10.1177/1098612X221077876 35266809 PMC9160961

[143] Marsilio S Pilla R Sarawichitr B Chow B Hill SL Ackermann MR. Characterization of the fecal microbiome in cats with inflammatory bowel disease or alimentary small cell lymphoma. Sci Rep. (2019) 9:19208. 10.1038/s41598-019-55691-w 31844119 PMC6914782

[144] Suchodolski JS Dowd SE Wilke V Steiner JM Jergens AE. 16s rRNA gene pyrosequencing reveals bacterial dysbiosis in the duodenum of dogs with idiopathic inflammatory bowel disease. PLoS ONE. (2012) 7:e39333. 10.1371/journal.pone.0039333 22720094 PMC3376104

[145] Suchodolski JS Markel ME Garcia-Mazcorro JF Unterer S Heilmann RM Dowd SE. The fecal microbiome in dogs with acute diarrhea and idiopathic inflammatory bowel disease. PLoS ONE. (2012) 7:e51907. 10.1371/journal.pone.0051907 23300577 PMC3530590

[146] Giaretta PR Rech RR Guard BC Blake AB Blick AK Steiner JM. Comparison of intestinal expression of the apical sodium-dependent bile acid transporter between dogs with and without chronic inflammatory enteropathy. J Vet Intern Med. (2018) 32:1918–26. 10.1111/jvim.15332 30315593 PMC6271328

[147] Minamoto Y Minamoto T Isaiah A Sattasathuchana P Buono A Rangachari VR. Fecal short-chain fatty acid concentrations and dysbiosis in dogs with chronic enteropathy. J Vet Intern Med. (2019) 33:1608–18. 10.1111/jvim.15520 31099928 PMC6639498

[148] Liu S Zhao W Lan P Mou X. The microbiome in inflammatory bowel diseases: from pathogenesis to therapy. Protein Cell. (2021) 12:331–45. 10.1007/s13238-020-00745-3 32601832 PMC8106558

[149] DeGruttola AK Low D Mizoguchi A Mizoguchi E. Current understanding of dysbiosis in disease in human and animal models. Inflamm Bowel Dis. (2016) 22:1137–50. 10.1097/MIB.0000000000000750 27070911 PMC4838534

[150] Del Carro A Corrò M Bertero A Colitti B Banchi P Bertolotti L. The evolution of dam-litter microbial flora from birth to 60 days of age. BMC Vet Res. (2022) 18:95. 10.1186/s12917-022-03199-3 35277176 PMC8915469

[151] Blake AB Cigarroa A Klein HL Khattab MR Keating T Van De Coevering P. Developmental stages in microbiota, bile acids, and clostridial species in healthy puppies. J Vet Intern Med. (2020) 34:2345–56. 10.1111/jvim.15928 33047396 PMC7694855

[152] Guard BC Mila H Steiner JM Mariani C Suchodolski JS Chastant-Maillard S. Characterization of the fecal microbiome during neonatal and early pediatric development in puppies. PLoS ONE. (2017) 12:e0175718. 10.1371/journal.pone.0175718 28448583 PMC5407640

[153] You I Kim MJ. Comparison of gut microbiota of 96 healthy dogs by individual traits: breed, age, and body condition score. Animals. (2021) 11:2432. 10.3390/ani11082432 34438891 PMC8388711

[154] Alessandri G Argentini C Milani C Turroni F Cristina Ossiprandi M van Sinderen D. Catching a glimpse of the bacterial gut community of companion animals: a canine and feline perspective. Microb Biotechnol. (2020) 13:1708–32. 10.1111/1751-7915.13656 32864871 PMC7533323

[155] Masuoka H Shimada K Kiyosue-Yasuda T Kiyosue M Oishi Y Kimura S. Transition of the intestinal microbiota of cats with age. PLoS ONE. (2017) 12:e0181739. 10.1371/journal.pone.0181739 28813445 PMC5558916

[156] Deusch O O'Flynn C Colyer A Swanson KS Allaway D Morris P. longitudinal study of the feline faecal microbiome identifies changes into early adulthood irrespective of sexual development. PLoS ONE. (2015) 10:e0144881. 10.1371/journal.pone.0144881 26659594 PMC4682054

[157] Hooda S Vester Boler BM Kerr KR Dowd SE Swanson KS. The gut microbiome of kittens is affected by dietary protein:carbohydrate ratio and associated with blood metabolite and hormone concentrations. Br J Nutr. (2013) 109:1637–46. 10.1017/S0007114512003479 22935193

[158] Melandri M Aiudi GG Caira M Alonge S. A biotic support during pregnancy to strengthen the gastrointestinal performance in puppies. Front Vet Sci. (2020) 7:417. 10.3389/fvets.2020.00417 32851009 PMC7417339

[159] Alonge S Aiudi GG Lacalandra GM Leoci R Melandri M. Pre- and probiotics to increase the immune power of colostrum in dogs. Front Vet Sci. (2020) 7:570414. 10.3389/fvets.2020.570414 33240949 PMC7681242

[160] Adogony V Respondek F Biourge V Rudeaux F Delaval J Bind JL. Effects of dietary scFOS on immunoglobulins in colostrums and milk of bitches. J Anim Physiol Anim Nutr. (2007) 91:169–74. 10.1111/j.1439-0396.2007.00688.x 17516936

[161] Mark-Christensen A Lange A Erichsen R Frøslev T Esen B Sørensen HT. Early-life exposure to antibiotics and risk for Crohn's disease: a nationwide Danish birth cohort study. Inflamm Bowel Dis. (2022) 28:415–22. 10.1093/ibd/izab085 34000050 PMC8889299

[162] Pan WH Sommer F Falk-Paulsen M Ulas T Best P Fazio A. Exposure to the gut microbiota drives distinct methylome and transcriptome changes in intestinal epithelial cells during postnatal development. Genome Med. (2018) 10:27. 10.1186/s13073-018-0534-5 29653584 PMC5899322

[163] Yu DH Gadkari M Zhou Q Yu S Gao N Guan Y. Postnatal epigenetic regulation of intestinal stem cells requires DNA methylation and is guided by the microbiome. Genome Biol. (2015) 16:211. 10.1186/s13059-015-0763-5 26420038 PMC4589031

[164] Guard BC Barr JW Reddivari L Klemashevich C Jayaraman A Steiner JM. Characterization of microbial dysbiosis and metabolomic changes in dogs with acute diarrhea. PLoS ONE. (2015) 10:e0127259. 10.1371/journal.pone.0127259 26000959 PMC4441376

[165] Pilla R Gaschen FP Barr JW Olson E Honneffer J Guard BC. Effects of metronidazole on the fecal microbiome and metabolome in healthy dogs. J Vet Intern Med. (2020) 34:1853–66. 10.1111/jvim.15871 32856349 PMC7517498

[166] Stavroulaki E Suchodolski JS Pilla R Fosgate GT Sung C-H Lidbury JA. Short-and long-term effects of amoxicillin/clavulanic acid or doxycycline on the gastrointestinal microbiome of growing cats. PLoS ONE. (2021) 16:e0253031. 10.1371/journal.pone.0253031 34910719 PMC8673677

[167] Schulfer AF Battaglia T Alvarez Y Bijnens L Ruiz VE Ho M. Intergenerational transfer of antibiotic-perturbed microbiota enhances colitis in susceptible mice. Nat Microbiol. (2018) 3:234–42. 10.1038/s41564-017-0075-5 29180726 PMC5780248

[168] Agrawal M Sabino J Frias-Gomes C Hillenbrand CM Soudant C Axelrad JE. Early life exposures and the risk of inflammatory bowel disease: systematic review and meta-analyses. EClinicalMedicine. (2021) 36:100884. 10.1016/j.eclinm.2021.100884 34308303 PMC8257976

[169] Suchodolski JS. Metabolic Consequences of Gut Dysbiosis in Dogs With IBD. Vancouver, BC: Nestlé Purina Companion Animal Nutrition (CAN) Summit (2017).

[170] Sacoor C Barros LM Montezinho L. What are the potential biomarkers that should be considered in diagnosing and managing canine chronic inflammatory enteropathies? Open Vet J. (2021) 10:412–30. 10.4314/ovj.v10i4.9 33614437 PMC7830176

[171] Maeda S Ohno K Fujiwara-Igarashi A Tomiyasu H Fujino Y Tsujimoto H. Methylation of TNFRSF13b and TNFRSF13c in duodenal mucosa in canine inflammatory bowel disease and its association with decreased mucosal IGA expression. Vet Immunol Immunopathol. (2014) 160:97–106. 10.1016/j.vetimm.2014.04.005 24814046

[172] Kathrani A Werling D Allenspach K. Canine breeds at high risk of developing inflammatory bowel disease in the South-Eastern UK. Vet Rec. (2011) 169:635. 10.1136/vr.d5380 21896567

[173] Dreschel NA. The effects of fear and anxiety on health and lifespan in pet dogs. Appl Anim Behav Sci. (2010) 125:157–62. 10.1016/j.applanim.2010.04.003

[174] Bray EE Sammel MD Cheney DL Serpell JA Seyfarth RM. Effects of maternal investment, temperament, and cognition on guide dog success. Proc Natl Acad Sci USA. (2017) 114:9128–33. 10.1073/pnas.1704303114 28784785 PMC5576795

[175] Gross B Garcia-Tapia D Riedesel E Ellinwood NM Jens JK. Normal canine brain maturation at magnetic resonance imaging. Vet Radiol Ultrasound. (2010) 51:361–73. 10.1111/j.1740-8261.2010.01681.x 20806866 PMC2936715

[176] Brydges NM. Pre-pubertal stress and brain development in rodents. Curr Opin Behav Sci. (2016) 7:8–14. 10.1016/j.cobeha.2015.08.003

[177] Dietz L Arnold A-MK Goerlich-Jansson VC Vinke CM. The importance of early life experiences for the development of behavioural disorders in domestic dogs. Behaviour. (2018) 155:83–114. 10.1163/1568539X-00003486

[178] Battaglia CL. Periods of early development and the effects of stimulation and social experiences in the canine. J Vet Behav. (2009) 4:203–10. 10.1016/j.jveb.2009.03.003

[179] Houpt KA Zicker S. Dietary effects on canine and feline behavior. Vet Clin North Am Small Anim Pract. (2003) 33:405–16, vii–viii. 10.1016/S0195-5616(02)00115-8 12701518

[180] Tiira K Lohi H. Early life experiences and exercise associate with canine anxieties. PLoS ONE. (2015) 10:e0141907. 10.1371/journal.pone.0141907 26528555 PMC4631323

[181] Guardini G Mariti C Bowen J Fatjó J Ruzzante S Martorell A. Influence of morning maternal care on the behavioural responses of 8-week-old Beagle puppies to new environmental and social stimuli. Appl Anim Behav Sci. (2016) 181:137–44. 10.1016/j.applanim.2016.05.006

[182] Foyer P Wilsson E Jensen P. Levels of maternal care in dogs affect adult offspring temperament. Sci Rep. (2016) 6:19253. 10.1038/srep19253 26758076 PMC4725833

[183] McMillan FD. Behavioral and psychological outcomes for dogs sold as puppies through pet stores and/or born in commercial breeding establishments: current knowledge and putative causes. J Vet Behav. (2017) 19:14–26. 10.1016/j.jveb.2017.01.001

[184] Casey RA Loftus B Bolster C Richards GJ Blackwell EJ. Human directed aggression in domestic dogs (*Canis familiaris*): occurrence in different contexts and risk factors. Appl Anim Behav Sci. (2014) 152:52–63. 10.1016/j.applanim.2013.12.003

[185] Wormald D Lawrence AJ Carter G Fisher AD. Analysis of correlations between early social exposure and reported aggression in the dog. J Vet Behav. (2016) 15:31–6. 10.1016/j.jveb.2016.08.071

[186] Freedman DG King JA Elliot O. Critical period in the social development of dogs. Science. (1961) 133:1016–7. 10.1126/science.133.3457.1016 13701603

[187] Lazarowski L Krichbaum S Waggoner LP Katz JS. The development of problem-solving abilities in a population of candidate detection dogs (*Canis familiaris*). Anim Cogn. (2020) 23:755–68. 10.1007/s10071-020-01387-y 32333134

[188] Bray EE Gruen ME Gnanadesikan GE Horschler DJ Levy KM Kennedy BS. Dog cognitive development: a longitudinal study across the first 2 years of life. Anim Cogn. (2021) 24:311–28. 10.1007/s10071-020-01443-7 33113034 PMC8035344

[189] Serpell JA Duffy DL. Aspects of juvenile and adolescent environment predict aggression and fear in 12-month-old guide dogs. Front Vet Sci. (2016) 3:49. 10.3389/fvets.2016.00049 27446937 PMC4916180

[190] Cimarelli G Virányi Z Turcsán B Rónai Z Sasvári-Székely M Bánlaki Z. Social behavior of pet dogs is associated with peripheral OXTR methylation. Front Psychol. (2017) 8:549. 10.3389/fpsyg.2017.00549 28443051 PMC5385375

[191] Simonson M. Effects of maternal malnourishment, development and behavior in successive generations in the rat and cat. In:Levitsky DA, editor. Malnutrition, Environment, and Behavior New Perspectives. Ithaca, NY: Cornell University Press (1979). p. 133-48.

[192] Gallo PV Werboff J Knox K. Protein restriction during gestation and lactation: development of attachment behavior in cats. Behav Neural Biol. (1980) 29:216–23. 10.1016/S0163-1047(80)90547-6 7387591

[193] Villablanca JR Olmstead CE. Neurological development of kittens. Dev Psychobiol. (1979) 12:101–27. 10.1002/dev.420120204 456750

[194] Turner DC. A review of over three decades of research on cat-human and human-cat interactions and relationships. Behav Processes. (2017) 141(Pt 3):297–304. 10.1016/j.beproc.2017.01.008 28119016

[195] McCune S. The impact of paternity and early socialization on the development of cats' behaviour to people and novel objects. Appl Anim Behav Sci. (1995) 45:109–24. 10.1016/0168-1591(95)00603-P

[196] Casey RA Bradshaw JWS. The effects of additional socialisation for kittens in a rescue centre on their behaviour and suitability as a pet. Appl Anim Behav Sci. (2008) 114:196–205. 10.1016/j.applanim.2008.01.003

[197] Bellamy KK Storengen LM Handegård KW Arnet EF Prestrud KW Overall KL. DRD2 is associated with fear in some dog breeds. J Vet Behav. (2018) 27:67–73. 10.1016/j.jveb.2018.07.008

[198] van Rooy D Haase B McGreevy PD Thomson PC Wade CM. Evaluating candidate genes OPRM1, DRD2, AVPR1a, and OXTR in Golden retrievers with separation-related behaviors. J Vet Behav. (2016) 16:22–7. 10.1016/j.jveb.2016.03.001

[199] van den Bergh BRH Dahnke R Mennes M. Prenatal stress and the developing brain: risks for neurodevelopmental disorders. Dev Psychopathol. (2018) 30:743–62. 10.1017/S0954579418000342 30068407

[200] Mourtzi N Sertedaki A Charmandari E. Glucocorticoid signaling and epigenetic alterations in stress-related disorders. Int J Mol Sci. (2021) 22:5964. 10.3390/ijms22115964 34073101 PMC8198182

[201] van den Bergh BRH van den Heuvel MI Lahti M Braeken M de Rooij SR Entringer S. Prenatal developmental origins of behavior and mental health: the influence of maternal stress in pregnancy. Neurosci Biobehav Rev. (2020) 117:26–64. 10.1016/j.neubiorev.2017.07.003 28757456

[202] Tyrka AR Ridout KK Parade SH. Childhood adversity and epigenetic regulation of glucocorticoid signaling genes: associations in children and adults. Dev Psychopathol. (2016) 28(4pt2):1319–31. 10.1017/S0954579416000870 27691985 PMC5330387

[203] Krol KM Moulder RG Lillard TS Grossmann T Connelly JJ. Epigenetic dynamics in infancy and the impact of maternal engagement. Sci Adv. (2019) 5:eaay0680. 10.1126/sciadv.aay0680 31663028 PMC6795517

[204] Franklin TB Russig H Weiss IC Gräff J Linder N Michalon A. Epigenetic transmission of the impact of early stress across generations. Biol Psychiatry. (2010) 68:408–15. 10.1016/j.biopsych.2010.05.036 20673872

[205] Jawaid A Jehle KL Mansuy IM. Impact of parental exposure on offspring health in humans. Trends Genet. (2021) 37:373–88. 10.1016/j.tig.2020.10.006 33189388

[206] Watkins AJ Ursell E Panton R Papenbrock T Hollis L Cunningham C. Adaptive responses by mouse early embryos to maternal diet protect fetal growth but predispose to adult onset disease. Biol Reprod. (2008) 78:299–306. 10.1095/biolreprod.107.064220 17989357

[207] Louwies T Johnson AC Orock A Yuan T Greenwood-Van Meerveld B. The microbiota-gut-brain axis: an emerging role for the epigenome. Exp Biol Med. (2020) 245:138–45. 10.1177/1535370219891690 31805777 PMC7016422

[208] Szyf M. The epigenetics of perinatal stress. Dialogues Clin Neurosci. (2019) 21:369–78. 10.31887/DCNS.2019.21.4/mszyf 31949404 PMC6952743

[209] Mohajeri MH La Fata G Steinert RE Weber P. Relationship between the gut microbiome and brain function. Nutr Rev. (2018) 76:481–96. 10.1093/nutrit/nuy009 29701810

[210] Zhu S Jiang Y Xu K Cui M Ye W Zhao G. The progress of gut microbiome research related to brain disorders. J Neuroinflammation. (2020) 17:25. 10.1186/s12974-020-1705-z 31952509 PMC6969442

[211] Sun C Velazquez MA Fleming TP. DOHaD and the periconceptional period, a critical window in time. In:Rosenfled CS, editor. The Epigenome and Developmental Origins of Health and Disease. New York, NY: Academic Press (2016). p. 33–47. 10.1016/B978-0-12-801383-0.00003-7

[212] Aiken CE Ozanne SE. Sex differences in developmental programming models. Reproduction. (2013) 145:R1–13. 10.1530/REP-11-0489 23081892

[213] Bock J Poeschel J Schindler J Börner F Shachar-Dadon A Ferdman N. Transgenerational sex-specific impact of preconception stress on the development of dendritic spines and dendritic length in the medial prefrontal cortex. Brain Struct Funct. (2016) 221:855–63. 10.1007/s00429-014-0940-4 25395153

[214] Laguna-Barraza R Bermejo-Álvarez P Ramos-Ibeas P de Frutos C López-Cardona AP Calle A. Sex-specific embryonic origin of postnatal phenotypic variability. Reprod Fertil Dev. (2012) 25:38–47. 10.1071/RD12262 23244827

[215] Tarrade A Panchenko P Junien C Gabory A. Placental contribution to nutritional programming of health and diseases: epigenetics and sexual dimorphism. J Exp Biol. (2015) 218(Pt 1):50–8. 10.1242/jeb.110320 25568451

[216] Sinclair KD Rutherford KM Wallace JM Brameld JM Stöger R Alberio R. Epigenetics and developmental programming of welfare and production traits in farm animals. Reprod Fertil Dev. (2016) 28:1443–78. 10.1071/RD16102 27439952

[217] Torres-Rovira L Tarrade A Astiz S Mourier E Perez-Solana M de la Cruz P. Sex and breed-dependent organ development and metabolic responses in foetuses from lean and obese/leptin resistant swine. PLoS ONE. (2013) 8:e66728. 10.1371/journal.pone.0066728 23935823 PMC3720837

[218] Vonnahme KA Hess BW Nijland MJ Nathanielsz PW Ford SP. Placentomal differentiation may compensate for maternal nutrient restriction in ewes adapted to harsh range conditions. J Anim Sci. (2006) 84:3451–9. 10.2527/jas.2006-132 17093240

[219] Murray JK Kinsman RH Lord MS Da Costa REP Woodward JL Owczarczak-Garstecka SC. 'Generation pup'—protocol for a longitudinal study of dog behaviour and health. BMC Vet Res. (2021) 17:1. 10.1186/s12917-020-02730-8 33397375 PMC7781182

[220] Chu ET Simpson MJ Diehl K Page RL Sams AJ Boyko AR. Inbreeding depression causes reduced fecundity in golden Retrievers. Mamm Genome. (2019) 30:166–72. 10.1007/s00335-019-09805-4 31115595 PMC6606663

[221] Guy MK Page RL Jensen WA Olson PN Haworth JD Searfoss EE. The golden Retriever lifetime study: establishing an observational cohort study with translational relevance for human health. Philos Trans R Soc Lond B Biol Sci. (2015) 370:20140236. 10.1098/rstb.2014.0230 26056371 PMC4581032

[222] Clements DN Handel IG Rose E Querry D Pugh CA Ollier WE. Dogslife: a web-based longitudinal study of Labrador Retriever health in the UK. BMC Vet Res. (2013) 9:13. 10.1186/1746-6148-9-13 23332044 PMC3559277

[223] Pugh CA Bronsvoort BMC Handel IG Querry D Rose E Summers KM. Incidence rates and risk factor analyses for owner reported vomiting and diarrhoea in Labrador Retrievers—findings from the DogsLife cohort. Prev Vet Med. (2017) 140:19–29. 10.1016/j.prevetmed.2017.02.014 28460746 PMC5424887

[224] Creevy KE Akey JM Kaeberlein M Promislow DEL. An open science study of ageing in companion dogs. Nature. (2022) 602:51–7. 10.1038/s41586-021-04282-9 35110758 PMC8940555

[225] Krontveit RI Nødtvedt A Sævik BK Ropstad E Skogmo HK Trangerud C. prospective study on canine hip dysplasia and growth in a cohort of four large breeds in Norway (1998–2001). Prev Vet Med. (2010) 97:252–63. 10.1016/j.prevetmed.2010.09.015 20956024

[226] Sævik BK Skancke EM Trangerud C. A longitudinal study on diarrhoea and vomiting in young dogs of four large breeds. Acta Vet Scand. (2012) 54:8. 10.1186/1751-0147-54-8 22300688 PMC3293024

[227] Murray JK Casey RA Gale E Buffington CAT Roberts C Kinsman RH. Cohort profile: the 'Bristol Cats Study' (BCS)—a birth cohort of kittens owned by UK households. Int J Epidemiol. (2017) 46:1749–50e. 10.1093/ije/dyx066 28645213

[228] Longstaff L Gruffydd-Jones TJ Buffington CT Casey RA Murray JK. Owner-reported lower urinary tract signs in a cohort of young cats. J Feline Med Surg. (2017) 19:609–18. 10.1177/1098612X16643123 27102690 PMC11128815

[229] Rowe E Browne W Casey R Gruffydd-Jones T Murray J. Risk factors identified for owner-reported feline obesity at around one year of age: dry diet and indoor lifestyle. Prev Vet Med. (2015) 121:273–81. 10.1016/j.prevetmed.2015.07.011 26265631

[230] Rowe EC Browne WJ Casey RA Gruffydd-Jones TJ Murray JK. Early-life risk factors identified for owner-reported feline overweight and obesity at around two years of age. Prev Vet Med. (2017) 143:39–48. 10.1016/j.prevetmed.2017.05.010 28622790

[231] Cat Phenotype and Health Information Registry (Cat PHIR) (2021). Available from: https://vgl.ucdavis.edu/cat-phir (accessed October 22, 2021).

[232] Rauluseviciute I Drabløs F Rye MB. DNA methylation data by sequencing: Experimental approaches and recommendations for tools and pipelines for data analysis. Clin Epigenetics. (2019) 11:193. 10.1186/s13148-019-0795-x 31831061 PMC6909609

[233] Yong WS Hsu FM Chen PY. Profiling genome-wide DNA methylation. Epigenetics Chromatin. (2016) 9:26. 10.1186/s13072-016-0075-3 27358654 PMC4926291

[234] Goyal D Limesand SW Goyal R. Epigenetic responses and the developmental origins of health and disease. J Endocrinol. (2019) 242:T105–t19. 10.1530/JOE-19-0009 31091503

[235] Megquier K Genereux DP Hekman J Swofford R Turner-Maier J Johnson J. Barkbase: epigenomic annotation of canine genomes. Genes. (2019) 10:433. 10.3390/genes10060433 31181663 PMC6627511

