# Supplementary material for: Environmental risk factors in puppies and kittens for developing chronic disorders in adulthood: A call for research on developmental programming
Source: Front Vet Sci. 2022 Dec 23;9:944821. doi: 10.3389/fvets.2022.944821 (PMC9816871; doi:10.3389/fvets.2022.944821)
Supplement: Supplementary file 1 [file Data_Sheet_1.PDF]

Supplementary Figure 1. Kinetics of embryo development. Timings are approximate estimates of *in vivo* events. Development stages at different times will vary by embryo and individual dam. Pink arrows denote events occurring in the oviduct; green and blue arrows denote events in the uterine horns. LH, luteinizing hormone.

a) Dog (1-5)

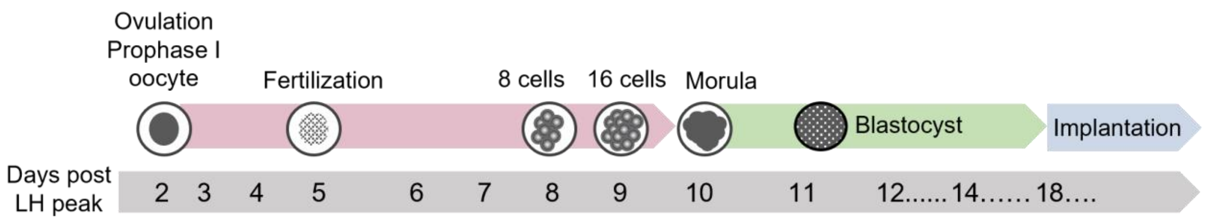

b) Cat (6, 7)

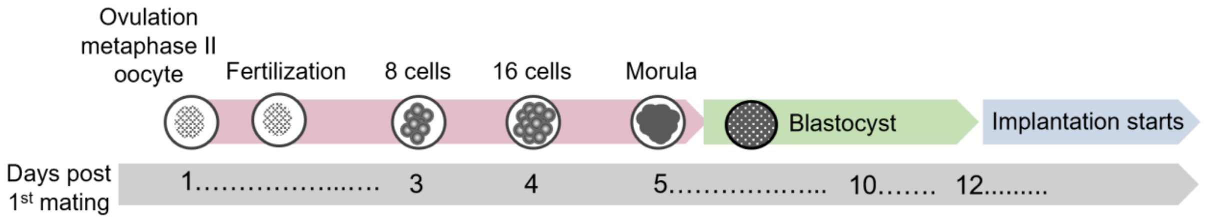

References Supplementary Figure 1

1. Abe Y, Suwa Y, Yanagimoto-Ueta Y, Suzuki H. Preimplantation development of embryos in Labrador Retrievers. *J Reprod Dev* (2008) 54(2):135-7. Epub 2008/01/17. doi: 10.1262/jrd.19139.

2. Chastant-Maillard S, Chebrout M, Thoumire S, Saint-Dizier M, Chodkiewicz M, Reynaud K. Embryo biotechnology in the dog: A review. *Reprod Fertil Dev* (2010) 22(7):1049-56. doi: 10.1071/rd09270.

3. Concannon PW. Reproductive cycles of the domestic bitch. *Anim Reprod Sci* (2011) 124(3-4):200-10. Epub 2010/11/09. doi: 10.1016/j.anireprosci.2010.08.028.

4. Reynaud K, Fontbonne A, Marseloo N, Thoumire S, Chebrout M, de Lesegno CV, et al. In vivo meiotic resumption, fertilization and early embryonic development in the bitch. *Reproduction* (2005) 130(2):193-201. doi: 10.1530/rep.1.00500.

5. Reynaud K, Fontbonne A, Marseloo N, Viaris de Lesegno C, Saint-Dizier M, Chastant-Maillard S. In vivo canine oocyte maturation, fertilization and early embryogenesis: A review. *Theriogenology* (2006) 66(6-7):1685-93. Epub 2006/02/20. doi: 10.1016/j.theriogenology.2006.01.049.

6. Denker HW, Eng LA, Mootz U, Hamner CE. Studies on the early development and implantation in the cat: 1. Cleavage and blastocyst formation. *Anat Anz* (1978) 144(5):457-68.

7. Swanson WF, Roth TL, Wildt DE. In vivo embryogenesis, embryo migration, and embryonic mortality in the domestic cat. *Biol Reprod* (1994) 51(3):452-64. doi: 10.1095/biolreprod51.3.452.

Supplementary Table 1. Overview of organogenesis and early development in dogs for selected organs. D, day; Mth, month; Wk, week; Yr, year.

**Period** 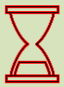	**Immune system** 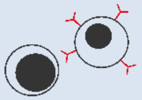	**Cardiovascular & musculoskeletal system** 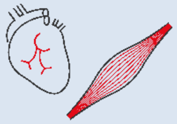	**Reproductive organs** 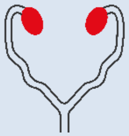	**Pituitary and adrenal glands and pancreas** 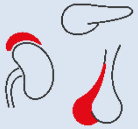	**Gut** 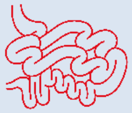	**Brain, neuroendocrine system, vision** 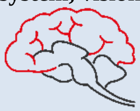
**Embryo Day 19–35** 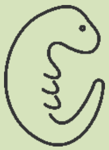	• D27–28: Primordia of spleen and thymus evident (1)	• D21–22: Primordial heart apparent (2)<br>• D21–24: Heart beat first visible (3)<br>• D22–25: Early development of limbs (2)<br>• D28:First ossification of skeleton observed (4)	• D22: Primordial germ cells detected in gonadal ridge (5)<br>• D22: Global DNA demethylation occurs in the male canine gonadal ridge (6)<br>• D21–25: Gonads remain undifferentiated (4)	• D25:Adeno-hypophysis first identifiable (7)<br>• D27: Primordial adrenal glands appear (7)<br>• D30: Pancreatic cells first detected – insulin +ve β-cells and α-cells (7)	• D22–25: Primitive gut established (2)	• D22–25: Brain vesicles (forebrain, midbrain and hind brain) have formed (2)<br>• D25–28: Retinal precursor formed (8)<br>• D29–32: Primitive brain still developing (2)
**Fetus Day 35 to birth** 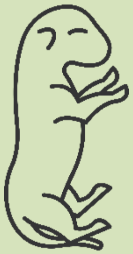	• D35–40: Thymus actively lymphopoietic (1)<br>• D45: Thymus microenvironment complete (1, 9)<br>• D45–52: Lymphocytes infiltrate lymph nodes (1)<br>• D45: Lymphocytes respond to mitogen (1, 9)<br>• D50: Thymocytes can respond to mitogen (1, 9)<br>• D45–52: Abundant hemopoietic stem cells in bone marrow (1)<br>• D45–55: Peyers patches present in small intestine (1)	• D35: Complete cardiovascular system apparent (2)<br>• D57: Last bones ossify (4)	• D35–40: Sex differentiation begins (5)<br>• Prespermatogonia present before birth (6)	• D38: Pituitary gland has same morphology as in adult dog (10)	• D44+: Differentiation of layers in gut wall detectable by ultrasound (11)	• D35: Metencephalon, optical source bridge and cerebellum forming (2)<br>• D35: Myelencephalon starting to form oblong medulla (2)

Supplementary Table 1 continued

**Birth to 8 weeks**<br><br>Timings relative to birth<br>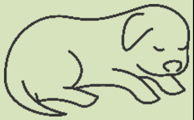	• At birth: Blood concentration of maternally derived antibodies close to zero (1, 12)<br>• At birth: functionally mature Peyer's patches (1)<br>• At birth a functional B and T cell system present (1)<br>• Day 1: Immunocompetent (post vaccination kinetics of antibodies similar to older puppies) (13)<br>• Days 0–21: Marked changes in proportions of different lymphocyte subsets (14)<br>(12)	• D18–21: Walking commences and is co-ordinated by D28 (15)<br>• Wk 4–6: Pronounced change from developmental to adult isoforms of myosin heavy chain in skeletal muscles (16)	• At birth: Ovary contains oogonia, but no follicles (17)<br>• Wk 2–3: First primordial follicles (17)<br>• Mth2: Germinal cells degenerated (female) (17)<br>• Wk 1: Uterine adenogenesis underway and prepubertal glandular proliferation complete by Wk6 (18)	• At birth: Adrenal capsule elements present but poorly demarcated (19)<br>• Wk8: global organization of pancreatic tissue mature (20)<br>• Wk8: Ratio of α:β cells is still less than in the adult (20)<br>• By Mth1: in adrenal gland demarcation between capsule and cortex evident and 3 cortical zones well defined (19)	• 16–24 hours: Gut closes to absorption of immunoglobulins (21)<br>• D1: GI tract colonized– aerotolerant bacteria dominant. Anaerobic bacteria subsequently increase in number and proportion (22)<br>• Wk 8: Microbiome changes significantly with age at least to Wk8 (23, 24)	• D16–36: Photoreceptors develop (8)<br>• Birth–Wk3: First cycle of postnatal myelinization of spinal cord (26)<br>• Wk4: Autonomic thermoregulation (15)<br>• By Wk4–5: Conditioned reflexes of vision and audition (26)<br>• By Wk6: Second cycle of postnatal myelinization (26)<br>• By Wk6: Neuronal development of sensory, motor, visual and auditory cortex relatively mature (26)<br>• Wk6: Brainstem and cerebellum have adult appearances (25)<br>• Wk8: Retina is mature (8)
**Post weaning**<br><br>Timings relative to birth<br>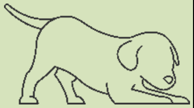	• Beyond Yr1: Proportion of lymphocyte populations continue to change (1)<br>• Mth2–3: Serum IgM levels attain adult levels (1)<br>• Mth6–9: Serum IgG levels approximate to adult levels (1)<br>• Mth12: Serum IgA levels attain adult levels (1)<br>• By Yr1: Serum immunoglobulin levels believed to reach adult values (12)<br>• Mth6–23: Thymus involution (12)	• By Mth2: Maturation of skeletal muscle fibres in terms of transition to adult myosin heavy chain isoforms nearly complete (16)<br>• Up to Yr2: Growth plates continue to close until up to 24 months (27)	• Mth4: Primary or early preantral follicles (6)<br>• Mth4 to Mth 5–6: Early antral follicles observed (6)<br>• Mth 6 onwards: Advanced antral follicles (6)<br>• ~Mth7: Spermatogenesis starts (~60-day cycles) (5)	• Up to Yr1: Marked morphological changes continue in adrenal gland (19)<br>• Age-related changes in chymotrypsin and amylase activity of pancreatic tissue apparent until adulthood (28)	• By Wk6: Greatest changes in gut microbiome have occurred (24) but changes still evident during Yr1 {You, 2021 #559}<br>• Gastric pepsin activity increases between D63 and adulthood (28)<br>• Apparent macronutrient digestibility increases until the end of growth (30)	• Learned responses such as delayed response develop after Wk8 (26)<br>• Wk 16: Corpus callosum and cerebrum have adult appearances (25)<br>• Neocortex myelinization continues until ~Mth9 (26)

References Supplementary Table 1

1. Felsburg PJ. Overview of immune system development in the dog: Comparison with humans. *Hum Exp Toxicol* (2002) 21(9-10):487-92. Epub 2002/12/03. doi: 10.1191/0960327102ht286oa.

2. Pieri N, Souza AF, Casals JB, Roballo K, Ambrósio CE, Martins DS. Comparative development of embryonic age by organogenesis in domestic dogs and cats. *Reprod Domest Anim* (2015) 50(4):625-31. Epub 2015/05/21. doi: 10.1111/rda.12539.

3. Siena G, Milani C. Usefulness of maternal and fetal parameters for the prediction of parturition date in dogs. *Animals (Basel)* (2021) 11(3). Epub 2021/04/04. doi: 10.3390/ani11030878.

4. Pretzer SD. Canine embryonic and fetal development: A review. *Theriogenology* (2008) 70(3):300-3. Epub 2008/06/03. doi: 10.1016/j.theriogenology.2008.04.029.

5. de Souza AF, de Ramos EC, Cury FS, Pieri NCG, Martins DS. The timeline development of female canine germ cells. *Repro Domest Anim* (2019) 54(7):964-71.

6. de Souza AF, Pieri NCG, Martins DDS. Step by step about germ cells development in canine. *Animals (Basel)* (2021) 11(3). Epub 2021/03/07. doi: 10.3390/ani11030598.

7. Thuróczy J. Foetal development of endocrine organs in dog. *Reprod Domest Anim* (2020) 55 Suppl 2:10-6. Epub 2020/06/11. doi: 10.1111/rda.13681.

8. Murphy CJ, Samuelson DA, Pollock RVH. Chapter 21: The eye. In: Evans HE, Delahunta A, editors. *Miller's Anatomy of the Dog*. 4 ed. St. Louis, Missouri, USA: Elsevier Saunders (2012). p. 746-85.

9. Day MJ. Small animal vaccination: A practical guide for vets in the UK. *In Practice* (2017) 39(3):110-8.

10. Sasaki F, Nishioka S. Fetal development of the pituitary gland in the Beagle. *Anat Rec* (1998) 251(2):143-51. Epub 1998/06/13. doi: 10.1002/(sici)1097-0185(199806)251:2<143::Aid-ar1>3.0.Co;2-#.

11. Gil EM, Garcia DA, Froes TR. In utero development of the fetal intestine: Sonographic evaluation and correlation with gestational age and fetal maturity in dogs. *Theriogenology* (2015) 84(5):681-6. Epub 2015/05/31. doi: 10.1016/j.theriogenology.2015.04.030.

12. Day MJ. Immune system development in the dog and cat. *J Comp Pathol* (2007) 137 Suppl 1:S10-5. Epub 2007/06/15. doi: 10.1016/j.jcpa.2007.04.005.

13. Chappuis G. Neonatal immunity and immunisation in early age: Lessons from veterinary medicine. *Vaccine* (1998) 16(14-15):1468-72. doi: 10.1016/s0264-410x(98)00110-8.

14. Faldyna M, Sinkora J, Knotigova P, Leva L, Toman M. Lymphatic organ development in dogs: Major lymphocyte subsets and activity. *Vet Immunol Immunopathol* (2005) 104(3-4):239-47. Epub 2005/03/01. doi: 10.1016/j.vetimm.2004.12.002.

15. Lawler DF. Neonatal and pediatric care of the puppy and kitten. *Theriogenology* (2008) 70(3):384-92. Epub 2008/06/03. doi: 10.1016/j.theriogenology.2008.04.019.

16. Strbenc M, Smerdu V, Pogacnik A, Fazarinc G. Myosin heavy chain isoform transitions in canine skeletal muscles during postnatal growth. *J Anat* (2006) 209(2):149-63. Epub 2006/08/02. doi: 10.1111/j.1469-7580.2006.00599.x.

17. Reynaud K, Fontbonne A, Saint-Dizier M, Thoumire S, Marnier C, Tahir MZ, et al. Folliculogenesis, ovulation and endocrine control of oocytes and embryos in the dog. *Reprod Domest Anim* (2012) 47 Suppl 6:66-9. Epub 2013/01/04. doi: 10.1111/rda.12055.

18. Cooke PS, Borsdorf DC, Ekman GC, Doty KF, Clark SG, Dziuk PJ, et al. Uterine gland development begins postnatally and is accompanied by estrogen and progesterone receptor expression in the dog. *Theriogenology* (2012) 78(8):1787-95. Epub 2012/09/11. doi: 10.1016/j.theriogenology.2012.05.028.

19. Hullinger RL. A histomorphological study of age changes in the canine adrenal gland. Retrospective Theses and Dissertations. 18317: Iowa State University of Science and Technology (1966).

20. Bricout-Neveu E, Pechberty S, Reynaud K, Maenhoudt C, José Lecomte M, Ravassard P, et al. Development of the endocrine pancreas in the Beagle dog: From fetal to adult life. *Anat Rec (Hoboken)* (2017) 300(8):1429-38. Epub 2017/03/16. doi: 10.1002/ar.23595.

21. Pereira M, Valério-Bolas A, Saraiva-Marques C, Alexandre-Pires G, Pereira da Fonseca I, Santos-Gomes G. Development of dog immune system: From in uterus to elderly. *Vet Sci* (2019) 6(4). Epub 2019/10/24. doi: 10.3390/vetsci6040083.

22. Buddington RK. Postnatal changes in bacterial populations in the gastrointestinal tract of dogs. *Am J Vet Res* (2003) 64(5):646-51. Epub 2003/05/21. doi: 10.2460/ajvr.2003.64.646.

23. Guard BC, Mila H, Steiner JM, Mariani C, Suchodolski JS, Chastant-Maillard S. Characterization of the fecal microbiome during neonatal and early pediatric development in puppies. *PLoS One* (2017) 12(4):e0175718. Epub 2017/04/28. doi: 10.1371/journal.pone.0175718.

24. Blake AB, Cigarroa A, Klein HL, Khattab MR, Keating T, Van De Coevering P, et al. Developmental stages in microbiota, bile acids, and clostridial species in healthy puppies. *J Vet Intern Med* (2020) 34(6):2345-56. Epub 2020/10/14. doi: 10.1111/jvim.15928.

25. Gross B, Garcia-Tapia D, Riedesel E, Ellinwood NM, Jens JK. Normal canine brain maturation at magnetic resonance imaging. *Vet Radiol Ultrasound* (2010) 51(4):361-73. Epub 2010/09/03. doi: 10.1111/j.1740-8261.2010.01681.x.

26. Fox MW. Overview and critique of stages and periods in canine development. *Dev Psychobiol* (1971) 4(1):37-54. Epub 1971/01/01. doi: 10.1002/dev.420040104.

27. Hammond G, McConnell F. Radiology of the appendicular skeleton. In: *BSAVA Manual of Canine and Feline Radiography and Radiology*. BSAVA Library (2013). p. 240-301.

28. Buddington RK, Elnif J, Malo C, Donahoo JB. Activities of gastric, pancreatic, and intestinal brush-border membrane enzymes during postnatal development of dogs. *Am J Vet Res* (2003) 64(5):627-34. Epub 2003/05/21. doi: 10.2460/ajvr.2003.64.627.

29. You I, Kim MJ. Comparison of gut microbiota of 96 healthy dogs by individual traits: Breed, age, and body condition score. *Animals (Basel)* (2021) 11(8). Epub 20210818. doi: 10.3390/ani11082432.

30. Weber M, Martin L, Biourge V, Nguyen P, Dumon H. Influence of age and body size on the digestibility of a dry expanded diet in dogs. *J Anim Physiol Anim Nutr (Berl)* (2003) 87(1-2):21-31. doi: 10.1046/j.1439-0396.2003.00410.x.

Supplementary Table 2. Overview of organogenesis and early development in cats for selected organs. D, day; Mth, month; Wk, week; Yr, year.

**Period** 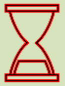	**Immune system** 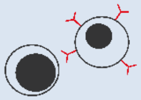	**Cardiovascular & musculoskeletal system** 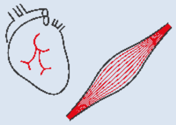	**Reproductive organs** 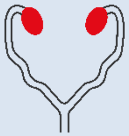	**Pituitary and adrenal glands and pancreas** 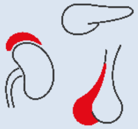	**Gut** 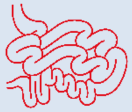	**Brain, neuroendocrine system, vision** 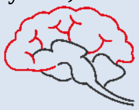
**Embryo**<br>**Day 19–35** 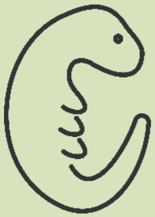	• D25: Lymphocytes in circulation (1)<br>• D25: Lymphocytes in circulation (1)<br>• D28–32: Lymphocytes scattered in developing lymph nodes (2)<br>• D32–38: First lymphoblasts in thymus (2)	• D18–19: Limb buds appear (2)<br>• D21: Primordial heart apparent (3)<br>• D22–25: Early development of limbs (3)<br>• D25–28: Lower limb starts perichondral ossification (2)	• D19–21: Genital ridge ventromedial of mesonephros (2)<br>• By Day 32: Testes and ovary are differentiated (2)	• D19–21: Pancreas primordia distinct (2)<br>• D23–25: Adrenal glands are differentiating (2)<br>• (2)D25–28: Adrenals produce corticoids (2)<br>• D25–28: Pancreas differentiates (2)<br>• D28–32: Adrenal cortex and medulla separated (2)<br>• D32–38: First pancreas islets (2)	• 16 hours: Immunoglobulin absorption stops (1)<br>• D22–25: Primitive gut established (3)<br>• D25–28: Gastric glands and intestinal villi form (2)	• D22–25: Brain vesicles (forebrain, midbrain and hind brain) formed (3)
**Fetus**<br>**Day 35 to birth** 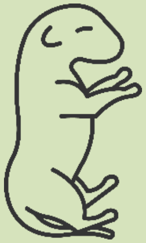		• D32: Complete cardiovascular system apparent (3)<br>• D34–38: skeletal muscle spindles have single primary myotubes with simple innervation (4)	• D38–44: Genitals are differentiated (2)			

Supplementary Table 2 continued

**Birth to 8 weeks**<br><br>Timings relative to birth<br>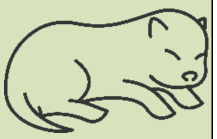	• At birth: Blood concentration of maternally derived antibodies close to zero (1, 5)<br>• At birth: Inability to mount substantial immune response to antigen (6)<br>• Wk6–12: Immunocompetence considered to be achieved but depends upon colostrum immunoglobulins ingested (1)<br>• D1 to at least Mth 3: proportions of some lymphocyte subsets change in serum, thymus and lymph nodes (6)	• By birth: Assembly of extra- and intrafusal skeletal muscle fibres (4)<br>• ~D31: Adequate standing and imperfect walking (7)<br>• ~D44: Normal walking and running (7)	• ~Mth 1: First primordial follicles appear (8)			• Birth–Mth3: All neurological functions mature progressively (7)<br>• D8–13: Spatial localization of auditory stimuli starts and complete by D12–20 (7)<br>• D10–15: Interactions with siblings, mother and/or objects, and play start (7)<br>• Day 26: Visual orienting observed (7)<br>• D30: Volumes of the neocortex, caudate nucleus, thalamus and substantia nigra are within adult ranges (9, 10)<br>• D47–83: Binocular coordination complete (7)<br>• D60: Volume of the red nucleus is within adult range (9)<br>• D70: Peak synaptic density in the visual cortex followed by significant decline (11)

**Post weaning**<br><br>Timings relative to birth<br>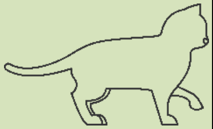	• Lymphocyte populations continue to change (6)<br>• Mth5–9: Secretory IgA from bile reaches adult levels (12)<br>• Mth 5+: Serum IgA reaches adult levels after Mth5 (13)<br>• Mth6–8: thymus starts to involute (1)				• By Wk 30–42: Gut microbiome has stabilized (14)	• Mth6: Volume of telencephalic white matter is close to adult volume (10)<br>• Mth3: Cerebral protein synthesis peaks in some brain regions (15)<br>• Mth6: local cerebral metabolic rate for glucose stabilizes to adult level (16)

References Supplementary Table 2

1. Day MJ. Immune system development in the dog and cat. *J Comp Pathol* (2007) 137 Suppl 1:S10-5. Epub 2007/06/15. doi: 10.1016/j.jcpa.2007.04.005.

2. Knospe C. Periods and stages of the prenatal development of the domestic cat. *Anat Histol Embryol* (2002) 31(1):37-51. Epub 2002/02/14. doi: 10.1046/j.1439-0264.2002.00360.x.

3. Pieri N, Souza AF, Casals JB, Roballo K, Ambrósio CE, Martins DS. Comparative development of embryonic age by organogenesis in domestic dogs and cats. *Reprod Domest Anim* (2015) 50(4):625-31. Epub 2015/05/21. doi: 10.1111/rda.12539.

4. Milburn A. Stages in the development of cat muscle spindles. *J Embryol Exp Morphol* (1984) 82:177-216. Epub 1984/08/01.

5. Felsburg PJ. Overview of immune system development in the dog: Comparison with humans. *Hum Exp Toxicol* (2002) 21(9-10):487-92. Epub 2002/12/03. doi: 10.1191/0960327102ht286oa.

6. Bortnick SJ, Orandle MS, Papadi GP, Johnson CM. Lymphocyte subsets in neonatal and juvenile cats: Comparison of blood and lymphoid tissues. *Lab Anim Sci* (1999) 49(4):395-400. Epub 1999/09/10.

7. Villablanca JR, Olmstead CE. Neurological development of kittens. *Dev Psychobiol* (1979) 12(2):101-27. Epub 1979/03/01. doi: 10.1002/dev.420120204.

8. Reynaud K, Saint-Dizier M, Fontbonne A, Thoumire S, Chastant-Maillard S. Follicle growth, oocyte maturation, embryo development, and reproductive biotechnologies in dog and cat. *Clin Theriogenology* (2020) 12(3):189-202.

9. Villablanca JR, Schmanke TD, Crutcher HA, Sung AC, Tavabi K. The growth of the feline brain from fetal into adult life. Ii. A morphometric study of subcortical nuclei. *Brain Res Dev Brain Res* (2000) 122(1):21-33. doi: 10.1016/s0165-3806(00)00047-x.

10. Villablanca JR, Schmanke TD, Lekht V, Crutcher HA. The growth of the feline brain from late fetal into adult life. I. A morphometric study of the neocortex and white matter. *Brain Res Dev Brain Res* (2000) 122(1):11-20. doi: 10.1016/s0165-3806(00)00046-8.

11. Winfield DA. The postnatal development of synapses in the visual cortex of the cat and the effects of eyelid closure. *Brain Res* (1981) 206(1):166-71. doi: 10.1016/0006-8993(81)90110-4.

12. Yamada T, Matsuda M, Ashida Y, Tsuchiya R, Wada Y, Matsubara T, et al. Isolation of secretory IgA from feline bile and bile IgA levels in growing cats. *J Vet Med Sci* (1992) 54(4):717-21. doi: 10.1292/jvms.54.717.

13. Yamada T, Nagai Y, Matsuda M. Changes in serum immunoglobulin values in kittens after ingestion of colostrum. *Am J Vet Res* (1991) 52(3):393-6.

14. Deusch O, O'Flynn C, Colyer A, Swanson KS, Allaway D, Morris P. A longitudinal study of the feline faecal microbiome identifies changes into early adulthood irrespective of sexual development. *PLoS One* (2015) 10(12):e0144881. Epub 2015/12/15. doi: 10.1371/journal.pone.0144881.

15. Hovda DA, Villablanca JR, Chugani HT, Barrio JR. Metabolic maturation of the brain: A study of local cerebral protein synthesis in the developing cat. *Brain Res* (2006) 1113(1):54-63. Epub 20060824. doi: 10.1016/j.brainres.2006.07.083.

16. Chugani HT, Hovda DA, Villablanca JR, Phelps ME, Xu WF. Metabolic maturation of the brain: A study of local cerebral glucose utilization in the developing cat. *J Cereb Blood Flow Metab* (1991) 11(1):35-47. Epub 1991/01/01. doi: 10.1038/jcbfm.1991.4.
